# Potential Therapeutic Options for COVID-19: Current Status, Challenges, and Future Perspectives

**DOI:** 10.3389/fphar.2020.572870

**Published:** 2020-09-15

**Authors:** Chandan Sarkar, Milon Mondal, Muhammad Torequl Islam, Miquel Martorell, Anca Oana Docea, Alfred Maroyi, Javad Sharifi-Rad, Daniela Calina

**Affiliations:** ^1^ Department of Pharmacy, Life Science School, Bangabandhu Sheikh Mujibur Rahman Science and Technology University, Gopalganj (Dhaka), Bangladesh; ^2^ Laboratory of Theoretical and Computational Biophysics, Ton Duc Thang University, Ho Chi Minh City, Vietnam; ^3^ Faculty of Pharmacy, Ton Duc Thang University, Ho Chi Minh City, Vietnam; ^4^ Department of Nutrition and Dietetics, Faculty of Pharmacy, and Centre for Healthy Living, University of Concepción, Concepción, Chile; ^5^ Universidad de Concepción, Unidad de Desarrollo Tecnológico, UDT, Concepción, Chile; ^6^ Department of Toxicology, University of Medicine and Pharmacy of Craiova, Craiova, Romania; ^7^ Department of Botany, University of Fort Hare, Alice, South Africa; ^8^ Phytochemistry Research Center, Shahid Beheshti University of Medical Sciences, Tehran, Iran; ^9^ Department of Clinical Pharmacy, University of Medicine and Pharmacy of Craiova, Craiova, Romania

**Keywords:** Severe Acute Respiratory Syndrome Coronavirus 2 (SARS-CoV-2), pandemic, COVID-19 proposed therapy, convalescent plasma, therapeutic challenges

## Abstract

The COVID-19 pandemic represents an unprecedented challenge for the researchers to offer safe, tolerable, and effective treatment strategies for its causative agent known as SARS-CoV-2. With the rapid evolution of the pandemic, even the off-label use of existing drugs has been restricted by limited availability. Several old antivirals, antimalarial, and biological drugs are being reconsidered as possible therapies. The effectiveness of the controversial treatment options for COVID-19 such as nonsteroidal antiinflammatory drugs, angiotensin 2 conversion enzyme inhibitors and selective angiotensin receptor blockers was also discussed. A systemic search in the PubMed, Science Direct, LitCovid, Chinese Clinical Trial Registry, and ClinicalTrials.gov data bases was conducted using the keywords “coronavirus drug therapy,” passive immunotherapy for COVID-19’, “convalescent plasma therapy,” (CPT) “drugs for COVID-19 treatment,” “SARS-CoV-2,” “COVID-19,” “2019-nCoV,” “coronavirus immunology,” “microbiology,” “virology,” and individual drug names. Systematic reviews, case presentations and very recent clinical guidelines were included. This narrative review summarizes the available information on possible therapies for COVID-19, providing recent data to health professionals.

## Introduction

**Figure 1:**
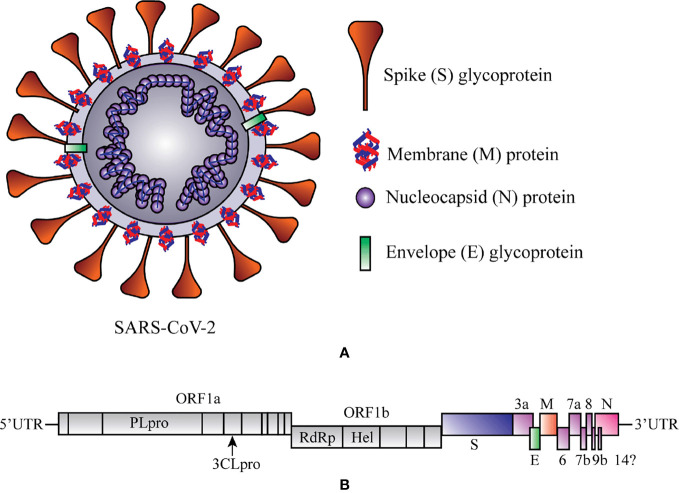
Schematic representation of structure and RNA genome of Severe Acute Respiratory Syndrome Coronavirus 2 (SARS-CoV-2). **(A)** Structure of SAR-CoV-2. **(B)** RNA genome sequence of SARS-CoV-2. 3CL^PRO^, 3-Chymotrypsin-like protease; Hel, helicase; ORF1a/b, Open reading frame 1a/b; PL^PRO^, Papain-like protease; RdRp, RNA-dependent RNA polymerase. At 5ʹ end 67% viral genome contains two open reading frames (ORF1a and ORF1b) that encode two significant replicase genes (*rep1a* and *rep1b*), and which helps to express large replicase polyprotein 1a/ab (pp1a and pp1ab) (Islam et al., 2020). These polyproteins produce nonstructural proteins (e.g., RNA-dependent RNA polymerase (RdRp) and helicase) after the cleavage with the help of two enzymes, papain-*like* cysteine protease (PL^PRO^) and 3-chymotrypsin-*like* serine protease (3CL^PRO^) (Zumla et al., 2016). At 3ʹ end 33% viral genome encodes the structural proteins (e.g., S, M, E, and N), which are required for the attachment of virus particle and entry of the viral genome into the host cell (Peiris et al., 2004).

The contemporary century has witnessed the outbreak of several corona viral intimidations that cause a spotlight on public health, education, economy, and travels and respond to the threat of a global pandemic. The ongoing viral infection is caused by SARS-CoV-2, which establishes a novel coronavirus disease 2019 (COVID-19). Analogous (79.6% similar) to SARS-CoV, the SARS-CoV-2 is one of the members of a relatively largest family of the RNA viruses and contains four important structural proteins, such as the surface spike (S) glycoprotein, membrane (M) protein, small envelope (E) glycoprotein, and the nucleocapsid (N) protein that help for its development completely (**Figure 1A**) (Schoeman and Fielding, 2019; Risitano et al., 2020). The positive-sense, single-stranded RNA genome of SARS-CoV-2 contains a cap at 5’ end and polyadenylated (A) sequence at 3’ end, serves as mRNA for replicase polyprotein translation (**Figure 1B**) (Wu et al., 2020).

From the beginning of the outbreak of SARS-CoV-2, it spreads immediately in most of the countries around the world and causes severe human diseases or death (Goumenou et al., 2020). The lack of effective drug therapy, and along with the high morbidity and mortality rates and its pandemic highlights the need for novel drug discovery for the treatment of COVID-19 (Tsatsakis et al., 2020).

Several national and international institutions and research groups are working collaboratively on a diversity of preemptive and beneficial interventions.

Cheap and widely available, dexamethasone is a steroid commonly used to treat allergic reactions, but also rheumatoid arthritis and asthma (Mititelu et al., 2020). British researchers who researched an effective treatment for COVID-19 reported that dexamethasone reduced deaths by a third among the most severely ill patients compared to regular treatment (Horby et al., 2020). It is currently conducting an analysis of the results obtained from the RECOVERY study arm regarding the use of dexamethasone-containing drugs in the treatment of hospitalized patients with COVID-19 infection. This component of the study looked at the effects of adding dexamethasone to regular therapeutic measures taken in adults who are being given invasive ventilation, those who are being given oxygen, or those who are not being given extra oxygen (Horby et al., 2020).

In the RECOVERY study, deaths occurred within 28 days of starting dexamethasone treatment. According to the preliminary results, in comparison with the routine measures, the administration of dexamethasone obtained the following: i) reduction by approximately 35% of the mortality rate in patients with invasive mechanical ventilation; ii) reduction by about 20% of the mortality rate in patients who were given oxygen without invasive ventilation; iii) nonreduction of the mortality rate in patients without oxygen therapy (Horby et al., 2020). As a result, in the UK, doctors have announced that patients will start receiving the first drug that has been shown to reduce COVID-19-associated death. While researchers believe that dexamethasone could save the lives of one in eight ventilator-connected patients, it has been shown to have few clinical benefits in less severe cases (Lu et al., 2020).

At least two major studies in the United States have shown that the antiviral drug remdesivir can reduce hospitalization period for patients with COVID-19. The results of these studies showed that remdesivir injections - originally intended as a treatment for Ebola - accelerated the patient’s recovery compared to placebo (Beigel et al., 2020; Goldman et al., 2020). Therefore, the US has authorized the emergency use of Remdesivir, an initiative followed by the European Union and several Asian nations, including Japan and South Korea (Gilead.Com, 2020).

China has completed clinical research on Favipiravir, an antiviral drug that has been shown to be clinically effective against the disease caused by the new coronavirus. Favipiravir, a flu medicine approved for clinical use in Japan in 2014, did not show any obvious side effects in the clinical trial (Heng et al., 2020).

More than 80 patients participated in the clinical trial, 35 of these patients received treatment with Favipiravir, and 45 were included in a control group. The results showed that patients treated with Favipiravir had negative results in testing for this virus in a shorter time compared to patients in the control group (Clinicaltrials.Gov, 2020a). Another randomized clinical trial also suggested that the therapeutic effect of Favipiravir was much better than that seen in the control group (Clinicaltrials.Gov, 2020bm). So, Favipiravir has been recommended by Chinese physicians and should be included in the diagnosis and treatment plan for COVID-19.

Prospective opportunities being reconnoitered include vaccine development, monoclonal antibodies (mAbs), interferon-based therapies, CPT, small-molecular drug therapies, and cell-based therapies (Li and De Clercq, 2020). (Calina et al., 2020).

In this comprehensive narrative review, we have sketched a current scenario on the most recent or ongoing clinical trials along with the remaining challenges and future perspectives of COVID-19 therapies.

## Methodology

Since there is little information about these drug candidates in the peer-reviewed literature, aimed at this review, we also collected data from the publicly available websites and electronic and print media. In order to obtain all registered therapeutic and preventative interventions under clinical investigation, a systemic search (up to 10^th^ June 2020) in the PubMed, Science Direct, LitCovid, Chinese Clinical Trial Registry, and ClinicalTrials.gov databases was conducted using the keywords “coronavirus drug therapy,” “passive immunotherapy for COVID-19,” “CPT,” “drugs for COVID-19 treatment,” “SARS-CoV-2,” “COVID-19,” “2019-nCoV,” “coronavirus immunology,” “microbiology,” “virology,” and individual drug names (**Table 1**). No language, country or study design restrictions were imposed. All information was evaluated in the knowledge about the treatment candidates, characteristics, dose/conc. (route of admin.), study systems, mechanism of action, and the stage of development of the COVID-19 therapies. The inclusion and exclusion criteria are given below.

**Table 1:** Potential Drugs against COVID-19 and their mechanisms of action.

Treatment candidates	Characteristics	Dose/route of administration	Study systems	Mechanism of action	Stage of development (Registry/Status)	References
**Antiviral drugs (nonspecific)**						
Immunoglobulin	Inhibitor of viral Fc receptor activation	0.5g/kg/day (iv)	Patients (n=80)	↓ antibody-dependent enhancement of infection↑endogenous Nabs	Phase 2 and 3(NCT04261426/Not yet recruiting)	(Cao et al., 2020; Clinicaltrials.Gov, 2020ah)
IFN-β1a	Cytokine signaling molecule	10 μg (iv)	Patients (n=7100)	↑cytoplasmic enzymes↓mRNA translation↓protein synthesis	Phase 4(NCT02735707/Recruiting)	(Clinicaltrials.Gov, 2020bf)
IFN- β1b	Cytokine in the interferon family	0.25 mg (sc)	Patients (n=80)	↑cytoplasmic enzymes↓mRNA translation a↓protein synthesis.	Phase 2(NCT04276688/Completed)	(Hung et al., 2020)
Interleukin-2	Cytokine signaling molecule	Low dose (IM)	Patients (n=20)	↑CD8+ T cells,↑CD4+ T,↑NK cell numbers.	Phase 1 (ChiCTR2000030167)	(Chictr.Org.Cn, 2020b)
CYNK-001	Cryopreserved allogeneic,off-the-shelf, placental-derived NK cell therapy	Multiple doses	Patients (n=86)	↑CD56+/CD3- NK cells	Phase 1 and 2(NCT04365101/Recruiting)	(Clinicaltrials.Gov, 2020av)
Baloxavir Marboxil	Cap snatching inhibitor	80 mg once a day (orally)	Patients (n=10)	↓viral cap-dependent endonuclease.	Clinical trial(ChiCTR 000029544)	(Lou et al., 2020)
**Antiviral drugs (broad spectrum)**						
Favipiravir	RNA polymerase inhibitor	1,800 and 600 mg	Patients (n=100)	↓RNA-dependent RNA polymerase (RdRp)	Phase 3(NCT04336904/Active, not recruiting)	(Furuta et al., 2017; Clinicaltrials.Gov, 2020l)
Arbidol	Direct antiviral/host-targeting agent	200 mg,tid or 400 mg, tid (orally)	Patients (n=500)	↓ membrane haemagglutinin fusion.	Phase 4(NCT04246242/Not yet recruiting)	(Smartpatients.Com, 2020)
Remdesivir	Adenosine analog	200 and 100 mg (IV)	Patients (n=237)	↓ SARS-CoV-2 replication↓RNA polymerase	Phase 3(NCT04257656/Terminated)	(Scavone et al., 2020; Wang et al., 2020b)
Galidesivir	Adenosineanalog	_	Patients (n=66)	↓viral RNA polymerase.	Phase 1(NCT03891420/Recruiting)	(Clinicaltrials.Gov, 2020bp)
**Antiviral drugs (antiretrovirals)**						
ASC09	Protease inhibitor	100 mg or 300 mg (orally)	Patients (n=160)	prevention of proteolytic cleavage.	Not Applicable (NCT04261907/Not yet recruiting)	(Clinicaltrials.Gov, 2020ak)
Azvudine	Nucleoside analog	1 mg (orally), 5 times daily	Patients (n=20)	↓reverse transcriptase →↓ replication of the virus.	Phase 3(ChiCTR2000029853)	(Chictr.Org.Cn, 2020e)
Danoprevir	Protease inhibitor	100 mg twice a day (orally)	Patients (n=11)	Danoprevir + ritonavir→↓transcription, ↓replication	Phase 4(NCT04291729/Completed)	(Chen et al., 2020)
Darunavir and Cobicistat	Protease inhibitor	Single dose (orally)	Patients (n=30)	Darunavir + cobicistat→↓ Cyt P-450 CYP3A.	Phase 3(NCT04252274/Recruiting)	(Clinicaltrials.Gov, 2020ab)
Lopinavir + Ritonavir	Protease inhibitor	100 and 400 mg (orally)	Patients (n=199)	↓metabolizing enzyme Cyt P450 3A by ritonavir↑ ½ life of lopinavir.	Clinical trial(ChiCTR2000029308)	(Cao et al., 2020; Dorward and Gbinigie, 2020)
**Antimalarial drug**						
Hydroxychloroquine	Antimalarial drug	200, 600, and 800 mg (orally)	Patients (n=3,000)	Hydroxychloroquine+Remdesivir →↓ viral replicationLopinavir/ritonavir + IF 1β →↓glycosylation of viral ACE-2.	Phase 3(NCT04308668/Recruiting)	(Clinicaltrials.Gov, 2020bb)
**Antibiotics and antiparasitics**						
Carrimycin	A polyether antibiotic	_	Patients (n=520)	Acts against Gram-positive bacteria, mycoplasma; fungi, and yeasts.	Phase 4(NCT04286503/Not yet recruiting)	(Clinicaltrials.Gov, 2020k)
Suramin sodium	Used to treat trypanosomiasis, onchocerciasis	_	Patients (n=20)	↓glycosylation of viral ACE-2 ↓quinone reductase 2.	Clinical trial(ChiCTR2000030029)	(Chictr.Org.Cn, 2020d)
Ivermectin	Used to treat parasitic infections	600 µg/kg (orally)	Patients (n=60)	↓replication of SARS-CoV-2.	Phase 2(NCT04374279/Not yet recruiting)	(Clinicaltrials.Gov, 2020bv)
Dihydroartemisinine + piperaquine	Inhibitor of viral Fc receptor activation.	Dihydroartemisinin + piperaquine (40 mg+320 mg) (orally)	Patients (n=40)	Interaction between its peroxide bridge and haem iron may underlie its antiviral action.	Phase 4(ChiCTR2000030082)	(Chictr.Org.Cn, 2020a)
Azithromycin	Macrolide antibiotic	500 mg (orally)	Patients (n=200)	It blocks internalization into host cells during the early phase of infection.	Phase 2(NCT04369365/Recruiting)	(Tran et al., 2019; Clinicaltrials.Gov, 2020bh)
Doxycycline	Semi-synthetic tetracycline antibiotic	200 mg/day (orally)	Patients (n=330)	↓ replication SARS-CoV-2↓IL-6 levels	Phase 3(NCT04371952/Not yet recruiting)	(Sargiacomo et al., 2020; Clinicaltrials.Gov, 2020y)
**Nonspecific antiinflammatory and immunosuppressive drugs**						
Corticosteroids	Immunomodulatingantiinflammatory	1mg/kg/day (IV)	Patients (n=86)	↓immune system↓inflammation↓ proinflammatory cytokines	Not Applicable(NCT04273321/Completed)	(Clinicaltrials.Gov, 2020aa)
Fingolimod	Immunosuppressant	0.5 mg once daily (orally)	Patients (n=30)	modulating S1P→sequesters lymphocytes in lymph nodes.	Phase 2(NCT04280588/Recruiting)	(Clinicaltrials.Gov, 2020al)
Leflunomide	DMARD and Immunosuppressant	300 mg once daily (orally)	Patients (n=20)	↓ dihydro-orotate dehydrogenase↓tyrosine kinases↓ intracellular transcription factors	Phase 1(NCT04361214/Recruiting)	(Clinicaltrials.Gov, 2020ar)
Thalidomide	Immunosuppressant and sedative drug	100 m (PO, QN)	Patients (n=100)	↓TNF-α↓ cell surface adhesion molecules involved in leukocyte migration.	Phase 2(NCT04273529/Not yet recruiting)	(Clinicaltrials.Gov, 2020ac)
Colchicine	Antiinflammatoy and antigout agents	0.5 mg (PO)	Patients (n=6,000)	↓microtubule assembly→↓inflammasome activation, ↓chemotaxis,↓leukotrienes, ↓cytokines,↓ phagocytosis.	Phase 3(NCT04322682/Recruiting)	(Niel and Scherrmann, 2006; Clinicaltrials.Gov, 2020m)
Ibuprofen	NSAID	200 mg	Patients (n=230)	↓COX,↓prostaglandins	Phase 4(NCT04334629/Recruiting)	(Cole and Frautschy, 2010; Clinicaltrials.Gov, 2020as)
Naproxen	NSAID	250 mg	Patients (n=584)	Inhibits the activity of cyclooxygenase enzymes.	Phase 3(NCT04325633/Not yet recruiting)	(Knights et al., 2010; Clinicaltrials.Gov, 2020ae)
Piclidenoson/CF101	Antiinflammatory drug	2 mg (orally)	Patients (n=40)	It binds to the Gi protein associated A3AR, ↓antiinflammatory effect↓IL-17, ↓ IL-23.	Phase 2(NCT04333472/Not yet recruiting)	(Clinicaltrials.Gov, 2020az)
**Kinase inhibitors**						
Jakotinib hydrochloride	JAK inhibitor	50 mg/bid(orally)	Patients (n=90)	↓AAK1↓JAK.	Phase 2(NCT04312594/Not yet recruiting)	(Zhang et al., 2020; Clinicaltrials.Gov, 2020ap)
Ruxolitinib	JAK inhibitor	5 mg (orally)	Patients (n=402)	↓protein tyrosine kinases↓JAK 1, ↓JAK 2↓inflammation↓cellular proliferation.	Phase 3(NCT04362137/Recruiting)	(Stebbing et al., 2020; Clinicaltrials.Gov, 2020ax)
Baricitinib	JAK inhibitor	4 mg/day(orally)	Patients (n=200)	Affinity for AP2-associated protein AAK1↓SARS-CoV-2 endocytosis.	Phase 2 and 3(NCT04320277/Not yet recruiting)	(Cantini et al., 2020; Clinicaltrials.Gov, 2020f)
Tofacitinib	JAK inhibitor	10 mg twice a day	Patients (n=50)	↓ JAKs↓phosphorylation, ↓activation of STATs	Phase 2(NCT04332042/Not yet recruiting)	(Clinicaltrials.Gov, 2020bs)
Imatinib	Kinase inhibitor	800 mg/day (orally)	Patients (n=99)	↓tumor growth of bcr-abl transfected murine myeloid cells as well as bcr-abl positive leukemia lines.	Phase 2(NCT04357613/Not yet recruiting)	(Moen et al., 2007; Clinicaltrials.Gov, 2020am)
**Monoclonal antibodies**						
Tozumab+adamumab	TNF-α inhibitor	_	Patients (n=60)	↓TNF-α↓ IL-6, ↓ IL-10	Phase 4(ChiCTR2000030580)	(Chictr.Org.Cn, 2020c)
Ravulizumab/ALXN1210	Component 5 (C5) inhibitor	Weight-based doses (IV)	Patients (n=270)	↓C5	Phase 3(NCT04369469/Not yet recruiting)	(Clinicaltrials.Gov, 2020ad)
Leronlimab/PA14/PRO-140	CCR5 antagonist	700 mg (SC)	Patients (n=390)	A humanized IgG4 and monoclonal antibody (mAb) to CCR5 →↓coronavirus entry, ↓viral infection of CD4 T-cells, ↓CCR5	Phase 2(NCT04347239/Recruiting)	(Clinicaltrials.Gov, 2020bo)
TJ003234	Anti-GM-CSF monoclonal antibody	3 and 6 mg/kg (IV)	Patients (n=144)	↑mAbs against GM-CSF.	Phase 1 and 2(NCT04341116/Recruiting)	(Clinicaltrials.Gov, 2020bl)
Nivolumab/Obtivo^®^	IgG4 monoclonal antibody	3 mg/kg (IV)	Patients (n=92)	It binds to the PD-L1 receptor and blocks its interaction with PD-L1 and PD-L2.	Phase 2(NCT04343144/Not yet recruiting)	(Wolchok et al., 2013; Clinicaltrials.Gov, 2020bu)
Meplazumab	Humanized mAb	10 mg (IV)	Patients (n=20)	It binds to IL-5 and prevents it from binding to its receptor.	Phase 1 and 2(NCT04275245/Recruiting)	(Bian et al., 2020)
Eculizumab	Recombinant humanized mAb	1,200 or 900 mg (IV)	Patients (n=120)	↓ C5 cleavage	Phase 2(NCT04346797/Recruiting)	(Clinicaltrials.Gov, 2020w)
Clazakizumab	Anti-IL- 6 monoclonal	25 mg (IV)	Patients (n=60)	↑IgG1 which binds to IL-6 and prevents its interaction and signaling *via* IL-6R.	Phase 2(NCT04348500/Recruiting)	(Clinicaltrials.Gov, 2020i)
Avdoralimab/IPH5401	Anti-C5aR antibody	Multiple doses (IV)	Patients (n=108)	blocks C5aR,↓inflammatory response in the lungs.	Phase 2(NCT04371367/Recruiting)	(Clinicaltrials.Gov, 2020e)
Lenzilumab	IgG1 kappa	IV infusion	Patients (n=238)	It targets CSF2/GM-CSF.	Phase 3(NCT04351152/Recruiting)	(Clinicaltrials.Gov, 2020ay)
LY3127804	A selective mAb	IV administration	Patients (n=200)	It acts against Angiopoietin 2 (Ang2).	Phase 2(NCT04342897/Recruiting)	(Clinicaltrials.Gov, 2020bj)
IFX-1	Antiinflammatories and Monoclonal antibody	Single dose/multiple doses (IV)	Patients (n=130)	↓C5a↓ Inflammation mediator modulators	Phase 2 and 3(NCT04333420/Recruiting)	(Clinicaltrials.Gov, 2020aw)
Gimsilumab/KIN-1901	Fully mAb	High dose on Day 1 & low dose on Day 8	Patients (n=270)	↓ GM-CSF.	Phase 2(NCT04351243/Recruiting)	(Clinicaltrials.Gov, 2020bn)
Actemra^®^/Tocilizumab	IL-6 inhibitor	800 mg (IV)	Patients (n=100)	↓ IL-6interrupts the process of CRS.	Phase 2(NCT04335071/Recruiting)	(Le et al., 2018; Clinicaltrials.Gov, 2020br)
Tocilizumab	IL-6 inhibitor (FDA granted)	800 mg (IV)	Patients (n=400)	↓ ILinterrupts the process of CRS.	Phase 2(NCT04317092/Recruiting)	(Fortes et al., 1973)
Kevzara^®^/Sarilumab	IL-6 inhibitor	200 and 400 mg	Patients (n=276)	↓immune response↓IL-6	Phase 2 and 3(NCT04315298/Recruiting)	(Arrytown and Paris, 2020)
Bevacizumab	Anti-VEGF monoclonal IgG1 antibody	7.5 mg/kg in 100 ml saline	Patients (n=130)	↓ viral proliferation,↓migration,↑IgG1	Phase 2(NCT04344782/Not yet recruiting)	(Kazazi-Hyseni et al., 2010; Clinicaltrials.Gov, 2020bt)
**Hormonal preparations**						
Aviptadil	Analog of VIP	50–150 pmol/kg/h (IV)	Patients (n=120)	↓NMDA-induced caspase-3 activation in the lung,↓IL6, ↓ TNFα	Phase 2(NCT04311697/Recruiting)	(Clinicaltrials.Gov, 2020ao)
Progesterone	Steroid hormone	100 mg (SC)	Patients (n=40)	↓inflammation↑repair of the respiratory epithelium.	Phase 1(NCT04365127/Recruiting)	(Hall and Klein, 2017; Clinicaltrials.Gov, 2020bc)
Sildenafil	PDE5 blocker	0.1 g/day (orally)	Patients (n=10)	↓cGMP/competitive binding at the phosphodiesterase binding site.	Phase 3(NCT04304313/Recruiting)	(Rogosnitzky et al., 2020; Clinicaltrials.Gov, 2020ba)
Triiodothyronine	Thyroid hormone	6 ml (IV)	Patients (n=60)	↓p38 MAPK activation↑tissue repair↑Akt activation	Phase 2(NCT04348513/Not yet recruiting)	(Pantos et al., 2020; Clinicaltrials.Gov, 2020bw)
Estradiol patch	Nuclear hormone	100 mg/day applied on the skin	Patients (n=110)	It interacts with a target cell receptor (Erα or Erβ) within the cytoplasm of the cell.	Phase 2(NCT04359329/Recruiting)	(Clinicaltrials.Gov, 2020aj)
**Cardiovascular drugs**						
Losartan	ACE2 receptor inhibitor	50 mg (orally)	Patients (n=200)	↓ vasoconstrictor and aldosterone-secreting effects of angiotensin II↓ binding of angiotensin II to the AT1 receptor.	Phase 2(NCT04312009/Recruiting)	(Tignanelli et al., 2020; Clinicaltrials.Gov, 2020au)
Valsartan	AT1R blockers (ARBs)	80 or 160 mg (orally)	Patients (n=651)	↓AT1R↑ACE2.	Phase 4(NCT04335786/Recruiting)	(Clinicaltrials.Gov, 2020bx)
Ramipril	ACE inhibitor	2.5 mg (orally)	Patients (n=560)	Inhibition of the renin-angiotensin system.	Phase 2(NCT04366050/Not yet recruiting)	(Clinicaltrials.Gov, 2020bd)
APN01	rhACE2	Single dose/multiple dose (IV)	Patients (n=200)	It mimics ACE2 - which is used by the virus to enter cells - acting as a decoy that binds to the virus and renders it inactive.	Phase 2 (NCT04335136/Recruiting)	(Taylor, 2020; Clinicaltrials.Gov, 2020bg)
Spironolactone	Antagonist of aldosterone	2 × 100 mg (orally)	Patients (n=60)	It acts through binding at the aldosterone-dependent sodium-potassium exchange site in the distal convoluted renal tubule.	Phase 4(NCT04345887/Not yet recruiting)	(Clinicaltrials.Gov, 2020bi)
**Blood and blood forming organs**						
Nafamostat Mesilate	Synthetic serine protease inhibitor and TMPRSS2-inhibitor.	IV administration	Patients (n=256)	↓thrombin,↓ factorXa, ↓ factor XIIa),↓kallikrein–kinin system,↓complement system, ↓pancreatic proteases.	Phase 2 and 3(NCT04352400/Not yet recruiting)	(Bittmann et al., 2020; Clinicaltrials.Gov, 2020ai)
Camostat Mesilate	TMPRSS2-inhibitor.	200 mg (orally)	Patients (n=114)	↓ viral replication	Phase 2(NCT04353284/Not yet recruiting)	(Clinicaltrials.Gov, 2020g)
**Vitamins or vitamin supplements**						
Vitamin C	Antioxidants	50 mg/kg (IV)	Patients (n=20)	↓inflammatory process↓development of respiratory failure requiring intubation.	Phase 1 and 2(NCT04357782/Recruiting)	(Clinicaltrials.Gov, 2020c)
		12g (IV)	Patients (n=140)	↓neutrophils accumulation in lung	Phase 2(NCT04264533/Recruiting)	(Clinicaltrials.Gov, 2020by)
		50 and 100 mg/kg (IV)	Patients (n=200)	↓ inflammatory process↓development of respiratory failure requiring intubation.	Phase 2(NCT04395768/Not yet recruiting)	(Clinicaltrials.Gov, 2020an)
Vitamin D	Immune modulator	50,000 and 400,000 IU (orally)	Patients (n=260)	↓ RAS↓lung damage	Phase 3(NCT04344041/Recruiting)	(Clinicaltrials.Gov, 2020x)
		50,000 IU once weekly(orally)	Patients (n=1,080)	↓ CAC.	Phase 2(NCT04363840/Not yet recruiting)	(Clinicaltrials.Gov, 2020aq)
		100.000 UI (orally)	Patients (n=1,265)	↓lung damage↓RAS	Phase 2(NCT04411446/Not yet recruiting)	(Clinicaltrials.Gov, 2020h)
Vitamin D3	Immuno-modulatory	100,000 IU(orally)	Patients (n=200)	↓ lung injury	Phase 2(NCT04400890/Not yet recruiting)	(Clinicaltrials.Gov, 2020be)

AP2, Adaptor protein-2; AAK1, Adaptor-associated kinase-1; ACE, Angiotensin converting enzyme; A3AR, A3 adenosine receptor; CRAC, Calcium release-activated calcium; CCR5, C-C chemokine receptor type 5; CSF2, colony stimulating factor 2; CRS, Cytokine release syndrome; C5, Complement 5; CAC, COVID-19-associated coagulopathy; DMARD, Disease-modifying antirheumatic drug; GM-CSF, Granulocyte-macrophage colony-stimulating factor; H-IG, Hyperimmune globulin; IV, Intravenous; IL-6, Interleukin-6; IgG4, Immunoglobulin G4; IM, Intramuscular; JAK, Janus-associated kinase; mAb, Monoclonal antibody; Nabs, Neutralizing antibodies; NRT, Nucleoside reverse transcriptase; NSAID, Nonsteroidal antiinflammatory drug; PO, Per oral; PDE, Phosphodiesterase enzyme; rhACE2, Recombinant human angiotensin-converting enzyme 2; RAS, Renin-angiotensin system; SC, Subcutaneous; SK2, Sphingosine kinase-2; S1P, Sphingosine 1-phosphate; TNF-α, Tumor necrosis factor alpha; TID, Three times a day; tPA, Tissue plasminogen activator; VEGF, Vascular endothelial growth factor; VIP, Vasoactive intestinal polypeptide.

### Inclusion Criteria

1. Studies on current COVID-19 drug therapy performed in SARS-CoV-2 infected patients;
2. Studies that exploited single and/or multiple animals;
3. Registered clinical trials on the proposed, repurposed or experimental candidates for the COVID-19 treatment that are recorded in online registries such as ClinicalTrials.gov and the International Clinical Trials Registry Platform (ICTRP) of the WHO;
4. Therapeutic candidates with beneficial consideration after clinical trials;
5. Therapeutic candidates exhibited auspicious effectiveness in contrast to COVID-19;
6. Studies with or without proposing mechanism of actions of the therapeutic candidates in COVID-19;
7. The most recent or ongoing clinical trial(s) on the individual treatment candidate.

### Exclusion Criteria

1. Data duplication, titles or abstracts not meeting the inclusion criteria;
2. Studies on antiviral drug candidates other than SARS-CoV-2;
3. Reports on treatment candidates that encode membrane (M), envelope (E), nucleocapsid (N) and spike (S) protein of genomic RNA other than SARS-CoV-2;
4. Active clinical trials were identified other than the ClinicalTrials.gov and the Chinese Clinical Trial Registry;
5. Previous clinical trial(s) on the individual candidate other than SARS-CoV-2 outbreak.

## Old and New Drugs Potentially Purposed for COVID-19 Treatment

**Figure 2:**
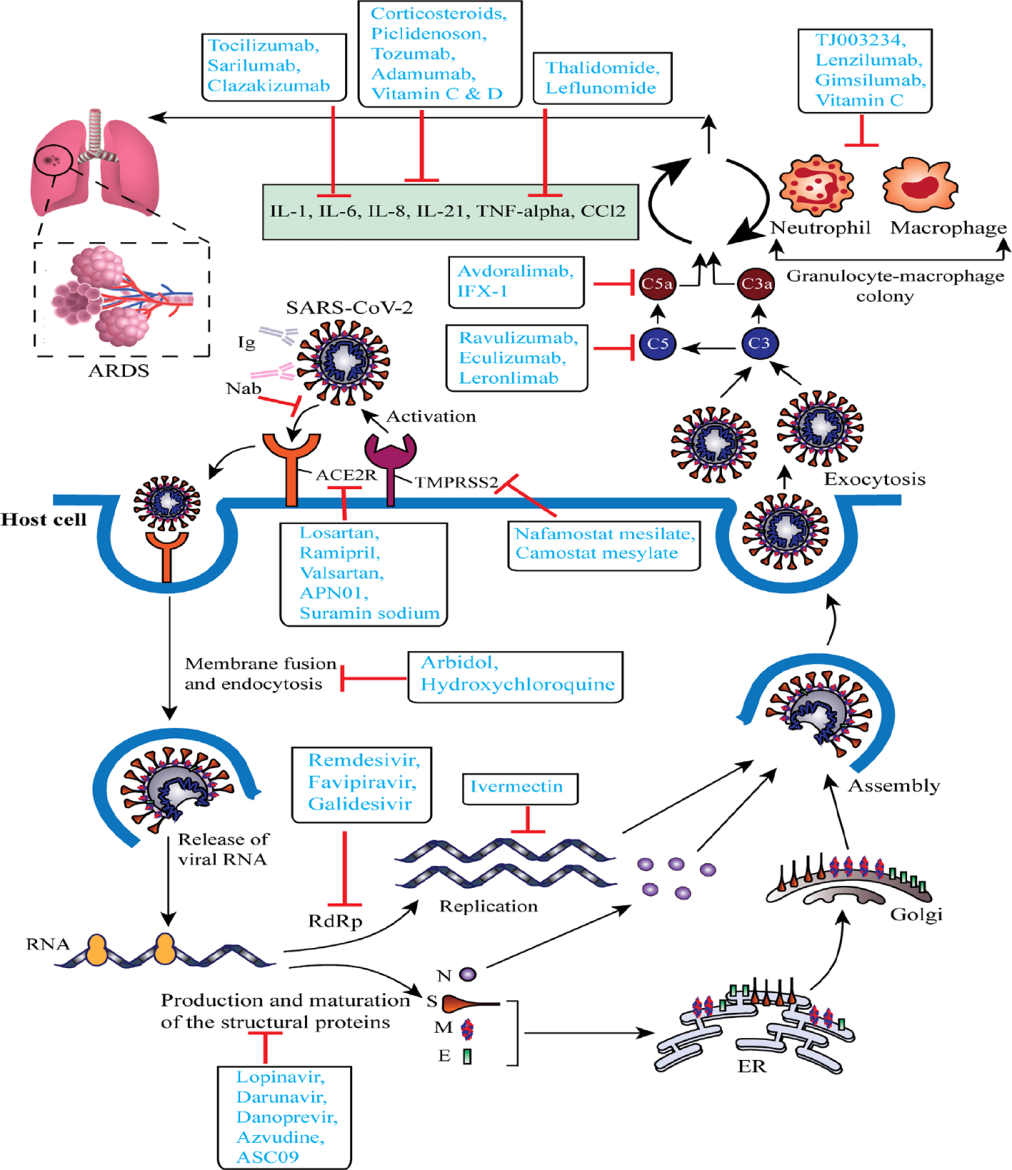
Schematic representation of virus-based treatment responses by targeting the Severe Acute Respiratory Syndrome Coronavirus 2 (SARS-CoV-2) replication cycle and SARS-CoV-2 associated acute respiratory distress syndrome. The proposed targets of most important candidates are noted. ACE2R, angiotensin-converting enzyme 2 receptor; ARDS, acute respiratory distress syndrome; ER, endoplasmic reticulum; E, envelope protein; Ig, immunoglobulin; M, membrane protein; N, nucleocapsid protein; Nab, neutralizing antibody; RdRp, RNA-dependent RNA polymerase; S, spike glycoprotein; TMPRSS2, type 2 transmembrane serine protease.

From December 2019, several clinical trials (including those not yet recruiting, recruiting, active, or completed) of the proposed or repurposed drugs in several countries around the world are continuously proceeding to deliver real-world clinical data for the COVID-19 challenges. We selected a total of 72 most current or ongoing clinical trials of the COVID-19 drug candidates with their mechanism of actions after refining through inclusion and exclusion criteria that might be helpful to screen, therefore, considered as starting points to discover and develop antiviral drug candidates for COVID-19 (**Table 1** and **Figure 2**).

**Table 1** shows i) antiviral drugs (nonspecific), ii) antiviral drugs (broad-spectrum), iii) antiviral drugs (antiretrovirals), iv) antimalarial drugs, v) antibiotics and antiparasitics, vi) nonspecific antiinflammatory and immunosuppressive drugs, vii) kinase inhibitors, viii) monoclonal antibodies, ix) hormonal preparations, x) cardiovascular drugs, and xi) blood and blood-forming organs.

### Antiviral Drugs (Nonspecific)

#### Immunoglobulin (Ig)

It is an inhibitor of viral fragment crystallizable (Fc) receptor activation, which prevents antibody-dependent enhancement of infection and provides boosting effects of endogenous neutralizing antibodies (Nabs) (Cao W. et al., 2020). Intravenous Ig is used to investigate to improve the treatment outcome of SARS-CoV-2 infection over the global pandemic with its capacity of proving passive immunity and antiinflammatory, and immunomodulatory effects. For this purpose, in phase 2/3 clinical trial (NCT04261426), 80 participants are treated with IV Ig at 0.5 g/kg/day dose for 5 days to understand the safety and efficacy of it in COVID-19 (Clinicaltrials.Gov, 2020ah).

#### Interferon (IFN)-β1a and -β1b

Interferon (IFN)-β1a is a cytokine signaling molecule used in the treatment of several chronic viral infections (e.g., HBV, HCV) that activates cytoplasmic enzymes, thereby, prevents mRNA translation and protein synthesis (Hensley et al., 2004; Clerico et al., 2007; Docea et al., 2016; Kamal et al., 2017). Recently, a research team of MJM Bontenprovides an adaptable research platform for the evaluation of treatment efficacy of IFN-β1a against the ongoing global pandemic in phase 4 clinical trial (NCT02735707) applying 10 μg intravenous (IV) dose once daily for 6 days in COVID-19 patients (n = 7,100) (Clinicaltrials.Gov, 2020bf). On the other hand, IFN-β1b, a cytokine used in the treatment of multiple sclerosis, is studied on 80 infected patients in phase 2 clinical trial (NCT04276688) with 0.25 mg subcutaneous (SC) dose for 3 days to evaluate the reduction of mortality rate (Hung et al., 2020). The combined therapy (lopinavir/ritonavir, ribavirin, and IFN-β1b) was found to suppress the viral load and reduce the mortality rate in the infected patients compared with the lopinavir/ritonavir (Clinicaltrials.Gov, 2020at).

#### Interleukin (IL)-2

It is another cytokine signaling molecule used in the immunotherapy treatment, especially in cancer (e.g., melanoma) (Rosenberg et al., 1994) and in the prevention of viral infection (e.g., HIV) (Kovacs et al., 1996; Boda et al., 2018). IL-2 is lymphocytotrophic hormone that is recognized and characterized as a fundamental for the generation and regulation of the immune response (Smith, 1988). It is a T lymphocyte product that stimulates T cells for the progression of the cell cycle *via* a finite number of interactions with its specific membrane receptors (Smith, 1988). A controlled phase 1 intervention (ChiCTR2000030167) increases the production of CD4+ T, CD8+ T and NK cell numbers in 20 infected patients at a low dose intramuscularly (IM) (Chictr.Org.Cn, 2020b).

#### CYNK-001

According to Celularity, the investigational new drug (IND) has been cleared by the authority of US Food and Drug Administration (FDA) for the use of CYNK-001 as an experimental allogeneic shelf cell (e.g., NK cell) therapy derived from the human placental CD34+ cells to treat COVID-19 patients (Celularity, 2020). Recently, a world-leading company “Celularity Incorporated” has experimented with the efficacy and safety of CYNK-001 (NCT04365101) on 86 participants, suggesting it has enriched for CD56+/CD3-NK cells (Clinicaltrials.Gov, 2020av).

#### Baloxavir Marboxil (S-033188)

Previously, baloxavir marboxil (S-033188) is used as a first-in-class antiviral prodrug that is converted as to its active form (baloxavir acid) through hydrolysis (Koshimichi et al., 2018) and in turn acts as a selective inhibitor of the cap-dependent endonuclease (Hayden et al., 2018) and the neuraminidase (NA) inhibitors (NAI) (O’hanlon and Shaw, 2019), which is specially approved for influenza. In a recent investigation (ChiCTR 2000029544), it is reported that the baloxavir marboxil selectively inhibits cap-dependent endonuclease of SARS-CoV-2 in 10 infected patients with 80 mg (once a day) oral dose (Lou et al., 2020).

### Antiviral Drugs (Broad-Spectrum, Inhibitors of RNA-Dependent RNA Polymerase)

#### Remdesivir (GS-5734)

Remdesivir (GS-5734), an approved HIV reverse transcriptase inhibitor, is a monophosphoramidate prodrug of an adenosine C-nucleoside with a similar chemical structure to the tenofovir alafenamide that consequently demonstrates as an active energetic C-adenosine nucleoside triphosphate analog and prevents RdRp as a broad-spectrum antiviral drug of several RNA viruses including as Coronaviridae and Flaviviridae (Agostini et al., 2018; Gordon et al., 2020; Ko et al., 2020). The first clinical use of remdesivir was for the treatment of Ebola. Based on the current pandemic, a report has recently been demonstrated an adaptive, randomized, placebo-controlled, double blind phase 3 (NCT04257656) clinical trial to evaluate the efficacy and safety of this drug (200 mg on day 1 and 100 mg once daily for 9 days) combined with the supportive care in the hospitalized 237 COVID-19 patients (Scavone et al., 2020; Wang et al., 2020b).

But this initial studies with remdesivir showed no benefit as underpowered (Davies et al., 2020; Wang et al., 2020a), this changed with the NIH study called COVID-19 Adaptive Treatment Trial (ACTT 3) in which the safety and efficacy of a treatment regimen consisting of remdesivir plus the interferon beta-1a immunomodulator in patients with coronavirus 2019 (COVID-19) will be evaluated (National Institutes of Health, 2020). Remdesivir has recently been granted a conditional marketing authorization in the European Union countries by the European Commission (Agency, 2020). A very recent study showed that this antiviral, originally developed against Ebola hemorrhagic fever, slightly reduces the recovery time of patients hospitalized with Covid-19 (15 to 11 days, on average). In contrast, this drug has not been shown to reduce mortality. The European Medicines Agency (EMA) has recommended the authorization of remdesivir (Velkury, Gilead Company) for patients infected with the new coronavirus, at EU level, by “conditional placing on the market.” The EMA has recommended the use of remdesivir in adults and adolescents over 12 years of age who have pneumonia and need oxygen supplementation in critically ill patients (Agency, 2020).

The FDA has also authorized the use of remdesivir in infection with the new SARS-CoV-2 coronavirus, through the Special Emergency Use Authorization (EUA). This approval allows doctors to administer remdesivir to patients with suspected or confirmed infection, severe form (have blood oxygen saturation SpO2 ≤ 94%, require oxygen therapy, mechanical ventilation or extracorporeal membrane-to-arterial oxygenation/ECMO), even outside of clinical trials. However, EUA is not a complete approval, as further studies are needed to confirm the effectiveness of this treatment. Urgent approval follows the publication of encouraging results from two studies involving remdesivir:

- Adaptive COVID-19 Treatment Trial (ACTT), organized by the US National Institute of Allergic and Infectious Diseases (NIAID): Phase III, randomized, placebo-controlled; 1,063 patients included; patients treated with remdesivir showed clinical improvement after a 31% shorter period; the study group had a median recovery time of 11 days, compared to 15 days in the control group; the study group had a mortality of 8%, compared to 11.6% in the control group (Health, 2020b).
- The SIMPLE study, organized by Gilead (the company producing remdesivir, veklury):

Phase III, without control group - patients receive a remdesivir treatment for 5 or 10 days;

Clinical improvement was similar in the two groups; half of the patients showed an improvement in the disease in the first 10 days, in the case of 5 days of treatment, and in the first 11 days (10 days of treatment); after 14 days, 60% of patients receiving remdesivir for 5 days were discharged, and 52.3% of those receiving 10 days were discharged (Gilead Sciences, 2020).

#### Favipiravir

Favipiravir (previously known as T-705 and Avigan) is a selective inhibitor of nonnucleoside RNA polymerase, which was developed and approved to treat influenza in Japan whereas it is already popular as a prodrug of purine nucleotide that is converted as to an active form namely favipiravir-ribofuranosyl-5′-triphosphate (RTP) by phosphoribosylation through the cellular enzymes (Furuta et al., 2013; Furuta et al., 2017). In response to the current global pandemic, with the help of a sponsor (Giuliano Rizzardini), a study (NCT04336904) on this drug is ongoing on 100 adult COVID-19 patients with 1800 mg/BID for day 1 and 600 mg/TID for day 2 and after that for a maximum of 14 days to evaluate the safety and efficacy of it combined with adequate supportive care (Clinicaltrials.Gov, 2020l).

#### Arbidol or Umifenovir

It is a selective broad-spectrum antiviral drug, which is initially licensed in Russia and China as a small indole-derivative molecule for the treatment of enveloped and nonenveloped virus infections (commonly influenza) through inhibiting the membrane haemagglutinin fusion (Blaising et al., 2014). Recently, a phase 4 clinical trial (NCT04246242) of arbidol is performed by the Xiangya Hospital of Central South University for determining the treatment efficacy and safety of it against the COVID-19 by applying the adaptable oral doses (e.g., 200 or 400 mg, TID) on 500 participants (Smartpatients.Com, 2020).

#### Galidesivir (BCX4430)

Galidesivir is another adenosine analog that demonstrates broad-spectrum antiviral activity against several types of viruses (e.g., togaviruses, filoviruses, arenaviruses, paramyxoviruses, orthomyxovirus bunyaviruses, CoVs, picornavirus, flaviviruses) and initially developed for the treatment of hepatitis C virus (HCV) (Westover et al., 2018). The first in-patient phase 1 clinical trial, is a randomized, placebo-controlled, and double-blind study to assess the safety, efficacy, pharmacokinetics, and tolerability of IV administration of galidesivir vs. placebo in hospitalized patients (n = 66) with either Group A (Yellow Fever) or Group B (COVID-19) (Clinicaltrials.Gov, 2020bp).

### Antiviral Drugs (Antiretrovirals, Protease Inhibitors)

#### Lopinavir/Ritonavir

Lopinavir/ritonavir, a US FDA approved co-formulated antiretroviral therapy for treating HIV protease, established selective *in vitro* antiviral activity against 3CL^PRO^ and PL^PRO^ proteases of the SARS-CoV-2 (Chu et al., 2004; De Wilde et al., 2014). Based on liver cytochrome P450 inhibition, the simultaneous use of ritonavir may upsurge the plasma half-life of lopinavir (Barragan and Podzamczer, 2008). More recently, it has been cited that an improved clinical outcome of patients (n = 199) with SARS-CoV-2 is appeared to be associated with a randomized open-label trial (ChiCTR2000029308) of orally administered lopinavir/ritonavir (100 and 400 mg) vs. standard care (Cao B. et al., 2020; Dorward and Gbinigie, 2020).

#### Azvudine

It is an experimental nucleoside analog that may inhibit the reverse enzyme transcriptase for viral transcription and show the potential against COVID-19 (Wang et al., 2014). Nucleoside analogs (e.g., azvudine, remdesivir, galidesivir) are the adenine or guanine derivatives that prevent the viral RNA synthesis and inhibit RdRp by encoding viral replication of several RNA viruses, including hCoVs (De Clercq, 2019). A phase 3 clinical trial (ChiCTR2000029853) on azvudine is ongoing at the People’s Hospital of Guangshan County to determine its better effectiveness against COVID-19 (Chictr.Org.Cn, 2020e).

#### Danoprevir

It is an orally available hepatitis C virus (HCV)NS3/4A protease inhibitor and recently approved for treating noncirrhotic genotype 1b chronic hepatitis C in China (Moucari et al., 2010). Danoprevir (100 mg/tablet) combined with ritonavir (100 mg/tablet) is currently in phase 4 clinical trial (NCT04291729) evaluating its safety and efficacy in COVID-19 patients (n = 11) and has shown the potential prevention against SARS-CoV-2 transcription and replication (Chen H. et al., 2020).

#### Darunavir

It is a US FDA approved nonpeptidic protease inhibitor (PI) for the treatment of HIV-1 infections, which is generally applied as a part of antiretroviral therapy (ART) together with a low boosting dose of ritonavir (Mckeage et al., 2009). The darunavir boosted with ritonavir (low dose) is swiftly absorbed and reaches peak plasma concentrations within 2.5–4 h (Rittweger and Arastéh, 2007). Darunavir is comprehensively and nearly absolutely metabolized by the hepatic cytochrome P450 (CYP) 3A4 enzymes (Rittweger and Arastéh, 2007). Patients with SARS-CoV-2 are being recruited in a randomized phase 3 clinical trial (NCT04252274) to evaluate the safety and efficacy of this drug and cobicistat (a potent human cytochrome P-450 3A (CYP3A) enzyme inhibitors used in the treatment of HIV/acquired immune deficiency syndrome (AIDS) infections) (Clinicaltrials.Gov, 2020ab).

#### TMC-310911(ASC09)

Structurally comparable to the darunavir, the TMC-310911 is a potent protease inhibitor that has been initially demonstrated for treating human immunodeficiency virus (HIV)-1 infections due to its proteolytic cleavage protection (Mina et al., 2020). Nowadays, several multinational companies are trying to develop the antiviral activity of this drug as a discerning agent against SARS-CoV-2 in combination with other HIV therapies, including ritonavir and lopinavir (Mina et al., 2020). Ascletis Pharmaceuticals Co., Ltd. is one of these multinational companies that provides information about an open-label trial (NCT04261907) of ASC09/ritonavir (300 mg/100 mg tablet) and lopinavir/ritonavir (200 mg/50 mg tablet), which are experimented on 160 participants to evaluate and compare their effectiveness in COVID-19 (Clinicaltrials.Gov, 2020ak).

### Antimalarial Drugs

The antimalarial drugs (e.g., hydroxychloroquine, chloroquine, quinacrine) are considered for a long time as effective therapies against malaria, and are also believed to have selective antiinflammatory effects against chronic inflammatory diseases (e.g., systemic lupus erythematosus, rheumatoid arthritis), have antiviral effects against different types of RNA viruses (e.g., dengue, chikungunya, HIV, SARS-CoVs, MERS-CoV), and also have immunomodulatory effects by inhibiting autophagy and lysosomal activity in host cells *via* cytokine signaling (Canadian Hydroxychloroquine Study Group, 1991; Rogoveanu et al., 2018; Vijayvargiya et al., 2020). A randomized and placebo-controlled phase 3 clinical study (NCT04308668) is being recruited for the treatment of COVID-19 patients (n = 3000) to evaluate the effectiveness of postexposure prophylaxis and preemptive therapy with hydroxychloroquine (200 mg tablet; 800 mg once, followed in 6 to 8 h by 600 mg, then 600 mg once daily for 4 days) (Clinicaltrials.Gov, 2020bb).

The World Health Organization recently announced that it is discontinuing clinical trials with hydroxychloroquine and the Lopinavir-Ritonavir combination due to failure to reduce mortality in patients infected with the novel coronavirus (WHO, 2020). Preliminary results of the SOLIDARITY study showed that hydroxychloroquine and the Lopinavir-Ritonavir combination reduced little or no mortality in hospitalized COVID-19 patients compared to the therapeutic standard (WHO, 2020). Also, according to RECOVERY, the first major clinical trial conducted by Oxford University in the UK, has been stopped because the delivered results, they showed that hydroxychloroquine has no beneficial effect on COVID-19 (Torjesen, 2020).

### Antibiotics and Antiparasitics

#### Carrimycin

On June 24, 2019, an interesting antibiotic (carrimycin) with a trade name of ‘Bite’ is originally developed for the treatment of upper respiratory infections approved by the country’s National Medical Products Administration in China (Trialsitenews, 2020).

A randomized (1:1), multicenter, open-controlled phase 3 clinical trial (NCT04286503) on 520 COVID-19 patients with carrimycin (experimental group) and lopinavir/ritonavir or arbidol or chloroquine phosphate (active comparator group) was launched by Beijing Youan Hospital to analysis the safety and efficacy of carrimycinin COVID-19 (Clinicaltrials.Gov, 2020k).

#### Suramin Sodium

Since the 1920s, suramin sodium (polysulfonated naphthylurea) has significantly been used to treat trypanosomiasis and onchocerciasis in humans and has also been seen as a potent inhibitor of reverse transcriptase enzyme of various types of retroviruses including HIV/AIDS, and various autocrine growth factors including tumor growth factor-beta (TGF-β), insulin-like growth factor I (IGF-I), platelet-derived growth factor (PDGF), epidermal growth factor (EGF), essential fibroblast growth factor (bFGF) and vascular endothelial growth factor (VEGF) (Hemady et al., 1996). It has also been used as an effective inhibitor of (Na^+^-K^+^)-activated ATPase and some hydrolytic and oxidative enzymes (Fortes et al., 1973). In retort to the ongoing pandemic, the hospitalized patients (n = 20) with proven SARS-CoV-2 infections at the First Affiliated Hospital of Zhejiang University School of Medicine are recruited to treat with it to evaluate its safety and efficacy in COVID-19 (Chictr.Org.Cn, 2020d).

#### Ivermectin

A macrocyclic lactone originally derived from an actinomycete (*Streptomyces avermitilis)* approved as a broad-spectrum antiparasitic and anthelmintic agent, is a 22,23-dihydro derivative of avermectin B1 with almost a similar structure to its naturally occurring precursor (abamectin) that is significantly used for the treatment of river blindness (onchocerciasis) and ectoparasitic disease, and also used against different types of nematode and arthropod parasites (Campbell et al., 1983; Campbell, 1985; Meinking et al., 1995). A randomized phase 2 (NCT04374279) clinical study has been applied in 60 severe COVID-19 patients to treat with standard care or standard care plus bicalutamide (150 mg once daily for 7 days) or ivermectin (600 µg/kg once daily for 3 days) (Clinicaltrials.Gov, 2020bv).

#### Dihydroartemisinine/Piperaquine

It is a fixed-dose combination antimalarial that contains 40 mg of dihydroartemisinin (potent and short-acting) and a 320 mg of partner drug, namely piperaquine (less-potent and long-acting) generally recommended by the World Health Organization (WHO) to treat uncomplicated malaria caused by *Plasmodium falciparum* (Amaratunga et al., 2016). On the other hand, the antiviral activity of dihydroartemisinin/piperaquine may underlie through interaction between its peroxide bridge and haem iron. Lately, a phase 4 clinical trial (ChiCTR2000030082) sponsored by the First Affiliated Hospital of Nanchang University is aimed to assess the anti-COVID-19 activity of this combination medicine on 40 COVID-19 patients (Chictr.Org.Cn, 2020a).

#### Azithromycin

The broad-spectrum antibiotic azithromycin is an orally administered acid-stableazalide antibacterial drug, which is an erythromycin derivative with a similar range of antimicrobial activity and developed pharmacokinetic physiognomies comparative to erythromycin (Peters et al., 1992; Zlatian et al., 2018). It is noted that the action of this drug is expanded significantly with a wide range of Gram-positive organisms, particularly *Haemophilus Influenza*-related with respiratory tract infections (Dunn and Barradell, 1996; Călina et al., 2017; Ungureanu et al., 2017). Most lately, azithromycin (500 mg) oral tablet has experimented as a prophylactic treatment following a randomized, single-blinded, placebo-controlled phase 2 trial (NCT04369365) in cancer patients (n = 200) undergoing antineoplastic therapy during the COVID-19 pandemic (Tran et al., 2019; Mohammad et al., 2020; Clinicaltrials.Gov, 2020bh).

#### Doxycycline

Doxycycline is a second-generation tetracycline that rapidly absorbed into the systemic circulation, distributed throughout the organism due to its function of lipophilicity, and eliminated through feces and urine (Saivin and Houin, 1988; Calina et al., 2016; Blejan et al., 2020). Xa study reports that doxycycline acts as a potent inhibitor of dengue viral replication and diminishesserum IL-6 levels at the time of viral infection (Sargiacomo et al., 2020). Patients (n = 330) with severe COVID-19 are recruited in a randomized, prospective, multicenter, double-blind phase 3 clinical study (NCT04371952) to evaluate the efficacy of doxycycline (200 mg/day) vs. a placebo (lactose 380 mg/capsule) (Clinicaltrials.Gov, 2020y).

### NonSpecific AntiInflammatory and Immunosuppressive Drugs

#### Corticosteroids

Glucocorticosteroid hormones corticosteroids are repeatedly used to treat acute respiratory distress syndrome (ARDS) and severe lung injury due to their capacity of diminishing inflammatory and fibrotic phenomena and defeating deposition of collagen (Claman, 1972). There also have some controversial for the therapeutic efficiency of corticosteroids despite the popularity of their administering. To study an anti-ARDS efficacy against COVID-19, 86 COVID-19 patients were treated in a randomized, prospective, and placebo-controlled fashion with methylprednisolone, 1 mg/kg/day (IV) for 7 days, or placebo (Clinicaltrials.Gov, 2020aa).

#### Fingolimod

A first-in-class orally administered compound fingolimod (FTY720) is a frequent immunology modulator of sphingosine-1-phosphate–a receptor that has exposed clinical efficacy and expansion on imaging in a nonrandomized phase 2 intervention (NCT04280588) against 30COVID-19 participants (Clinicaltrials.Gov, 2020al). It is initially used in multiple sclerosis thanks to its function of sequestering lymphocytes in lymph nodes (Chun and Hartung, 2010).

#### Leflunomide

FDA approved immunomodulatory prodrug leflunomide to treat rheumatoid arthritis as a disease-modifying antirheumatic drug that is rapidly converted to its active metabolite (A771726) after oral administration (Fox, 1998; Prakash and Jarvis, 1999). The immunosuppressant leflunomide causes inhibition of dihydro-orotate dehydrogenase and tyrosine kinases and degradation of intracellular transcription factors (Rozman, 2002). In order to find out the tolerability of this drug with a high dose (300 mg once daily), the University of Chicago recruited a single-center tolerability phase 1 clinical trial (NCT04361214) with leflunomide in the ambulatory patients (n = 20) with mild COVID-19 (Clinicaltrials.Gov, 2020ar).

#### Thalidomide

Firstly, the CIBA pharmaceutical company manufactured thalidomide in 1954 thanks to the prescribed drug as a sedative, antiemetic, and tranquillizer for the morning sickness (Franks et al., 2004). Then it is profoundly marketed and endorsed throughout the world due to having its multi-purposes functions such as antiangiogenesis, antifibrotic, immune regulation effects, and antiinflammatory (Shannon et al., 2008). It inhibits excess production of tumor necrosis factor-alpha (TNF-α) and suppresses the leukocyte migration. A research group of the First Affiliated Hospital of Wenzhou Medical University randomly allocated 100 patients to receive thalidomide (100 mg, orally for 14 days) or placebo at the same dose of thalidomide in a first prospective, multi-center, placebo-controlled, double-blind phase 2 intervention (NCT04273529) to evaluate the safety and efficacy of this drug in COVID-19 (Clinicaltrials.Gov, 2020ac).

#### Colchicine

It is an alkaloid derivative derived from a plant source, namely *Colchicum autumnale* (Liliaceae) (Terkeltaub, 2009). Colchicine is standing with a long history for its application in inflammatory diseases, including familial Mediterranean fever, and severe gout and Behçet’s disease (Niel and Scherrmann, 2006). In retort to the COVID-19, a research team of the Montreal Heart Institute assigned 6,000 COVID-19 patients to be given either colchicine or placebo (1:1 allocation ratio) for 30 days in a randomized, multi-center, double-blind, placebo, parallel controlled phase 3 clinical study to examine the reduction of mortality rate and lung difficulties associated with COVID-19 (Clinicaltrials.Gov, 2020m).

#### Ibuprofen

It is a nonsteroidal antiinflammatory drug (NSAID) involved in the class of 2 aryl propionic acid (2-APA) that was first announced in England in 1967 (Davies, 1998). It inhibits the production of prostaglandins by decreasing the activity of the enzyme cyclooxygenase (Cole and Frautschy, 2010; Rogoveanu et al., 2018). Ibuprofen is also familiar for the advanced treatment of osteoarthritis, rheumatoid arthritis, ankylosing spondylitis, gout, and Bartter’s syndrome (Kantor, 1979; Mititelu et al., 2020; Salehi et al., 2020). After registration (NCT04334629), the King’s College London initiated a phase 4 clinical trial (multicenter, randomized, controlled trial) of this drug at a daily dose of 200 mg in 230 patients to examine the reduction in the austerity and advancement of lung difficulties associated with COVID-19 (Clinicaltrials.Gov, 2020as).

#### Naproxen

Stereochemically naproxen is a potent nitric oxide‐releasing NSAID that is usually administered orally or rectally for the treatment of severe rheumatic disease and several nonrheumatic circumstances (Todd and Clissold, 1990). A study has been reported that a nitroxybutyl ester derivative of naproxen shows the less ulcerogenic in the gastrointestinal tract (GIT) than its mother NSAID (Davies et al., 1997). A randomized phase 3 clinical trial (NCT04325633) is established at Assistance Publique - Hôpitaux de Paris in hospitalized patients (n=584)with severe COVID-19 to treat with the standard of care plus naproxen(250 mg BID) and lansoprazole (30 mg daily) in order to determine the effectiveness of this drug in COVID-19 (Knights et al., 2010; Clinicaltrials.Gov, 2020ae).

#### Piclidenoson (CF101)

According to the report from the website of Can-Fite BioPharma, piclidenoson, commonly known as IB-MECA (methyl 1-[N6-(3-iodobenzyl)-adenin-9-yl]-b-D-ribofuronamide) is an active antiinflammatory agent that has been experimented in different types of experimental models. It acts after binding to the G protein associated A3AR, which induces a robust antiinflammatory effect by inhibiting IL-17 and -23.Patients (n = 40) with COVID-19 are assigned in an open-label, randomized, control phase 2 clinical trial (NCT04333472) to receive either piclidenoson (2 mg Q12H orally) with standard care as an experimental arm or standard care alone as a control arm (1:1 allocation ratio) on empty stomach of patients to evaluate the safety and efficacy against COVID-19 (Clinicaltrials.Gov, 2020az).

### Kinase Inhibitors

#### Jakotinib Hydrochloride

Jakotinib hydrochloride, an AP2-associated protein kinase 1 (AAK1) inhibitor as well as a Janus kinase (JAK) inhibitor, was recommended a conceivable candidate, making an allowance for its high rate of persistent virological response in COVID-19 patients (Zhang et al., 2020). To determine the antiviral and antiinfective activity of this drug (50 mg/BID, orally), a randomized phase 2 clinical intervention (NCT04312594) sponsored by Suzhou Zelgen Biopharmaceuticals Co., Ltd is assigned in 90 COVID-19 patients with idiopathic pulmonary fibrosis (Clinicaltrials.Gov, 2020ap).

#### Ruxolitinib

Ruxolitinib, formally known as INC424 or INCB18424, is a US FDA approved orally bioavailable JAK1/2 inhibitor usually used in the treatment of myelofibrosis as an effective and discerning inhibitor (Harrison et al., 2012; Stebbing et al., 2020). Ruxolitinib, a more auspicious repurposed antiviral agent to examine its safety and efficacy against randomized patients (n = 402) with COVID-19 a multicenter, double-blind, controlled, phase 3 clinical intervention (NCT04362137) has been recruited to treat with either ruxolitinib at a dose of 5 mg/BID plus standard of carein 2:1 allocation ratio or oral matching-image placebo plus standard of care for 14 days (Clinicaltrials.Gov, 2020ax).

#### Baricitinib

Baricitinib, orally bioavailable, is another potent and selective inhibitor of AAK1 and JAK1/2 (Richardson et al., 2020). It is a more auspicious repurposed antiviral agent with a unique mechanism of action targetingAAK1 and JAK1/2, and reducing SARS-CoV-2 endocytosis through binding to the cyclin g-associated kinase (GAK) (Cantini et al., 2020). Treatment with this drug is accompanied with a high rate of continuous virological response in patients (n = 200) with mild to moderate SARS-CoV-2 infection in the response-guided nonrandomized, prospective, open-label, 2-week, phase 2 and 3 interventions (NCT04320277) conducted in the Fabrizio Cantini, Hospital of Prato (Clinicaltrials.Gov, 2020f).

#### Tofacitinib

Tofacitinib, a persuasive oral inhibitor of the JAK1/2/3 (family: kinases), can alleviate alveolar inflammation thorough blocking interleukins signal such as IL-2, -4, -6, -7, -9, -15, and -21, which is generally approved as an immunomodulator and disease-modifying therapeutic agent for rheumatoid arthritis (Fleischmann et al., 2012; Sandborn et al., 2012). To examine the primary outcome of this drug in COVID-19, a single group assignment, prospective cohort, phase 2 study (NCT04332042) is being arranged by the Armando Gabrielli, Università Politecnica delle Marche to treat SARS-CoV-2 related interstitial pneumonia in patients (n = 50) with it at a dose of 10 mg/BID for 14 days (Clinicaltrials.Gov, 2020bs).

#### Imatinib

Imatinib, an approved agent for chronic myelogenous leukemia (CML) and gastrointestinal stromal tumor (GIST), can potentially inhibit the fusion protein Bcr-Abl and platelet-derived growth factor receptors (e.g., PDGFRα and PDGFRβ) (Peng et al., 2005; Moen et al., 2007). To study the antiviral effect of this drug, a research team of the Versailles Hospital randomly assigned 99 patients with nonsevere COVID-19 in phase 2, randomized, open-label, parallel clinical trial (NCT04357613), in a 1:1 ratio, to receive imatinib (800 mg/day) or standard therapy (Clinicaltrials.Gov, 2020am).

### Monoclonal Antibodies

#### Tozumab/Adamumab

Tozumab is used as an immunotherapy for the treatment of bilateral lung lesions, whereas adamumab is used in rheumatoid arthritis (Ying et al., 2020). A combination therapy (tozumab combined with adamumab) is applied in severe and critical COVID-19 patients (n = 60) having pneumonia in phase 4, randomized, single-center, prospective, controlled parallel trial (ChiCTR2000030580) to evaluate its safety and efficacy in COVID-19 (Chictr.Org.Cn, 2020c).

#### Ravulizumab (Ultomiris or ALXN1210)

Ravulizumab (also called Ultomiris and ALXN1210), a humanized monoclonal antibody, is firstly manufactured by the Alexion Pharmaceuticals as a new inhibitor of complement C5 for paroxysmal nocturnal haemoglobinuria (PNH) and atypical hemolytic uremic syndrome (aHUS) treatment (Mckeage, 2019). It was first approved intravenous drug for paroxysmal nocturnal hemoglobinuria (PNH) in USA in December 2018, which is developed from eculizumab to have a considerably higher terminal half-life (Röth et al., 2018). To determine the safety and efficacy of its in COVID-19,270 patients with severe pneumonia are randomly assigned to receive weight-based doses of ravulizumab (intravenously on Days 1, 5, 10, and 15) with the best supportive care by applying phase 3 open-label, randomized, controlled study (NCT04369469) (Clinicaltrials.Gov, 2020ad).

#### Leronlimab

FDA approved CCR5 (G protein-coupled receptor) antagonist, leronlimab (also called PA14 and PRO-140) is a humanized IgG4 and a monoclonal antibody (mAb) to CCR5 that significantly prevents CoV entry and inhibits viral infection of CD4 T-cells by blocking the CCR5 (Clinicaltrials.Gov, 2020bo). The unique mechanism of binding to CCR5, leronlimab may improve the activities of DDR-based treatments for different types of cancer (e.g., prostate, pancreatic, breast, colon, and melanoma), permitting the reduction in dose of standard chemotherapy (Pestell et al., 2020). Researchers in CytoDyn, Inc. designed a randomized, double blind, adaptive, placebo controlled phase 2b/3 clinical trial (NCT04347239) to assess the efficacy, safety, and tolerability of subcutaneous leronlimab (weekly doses of 700 mg) in 390patients with severe COVID-19 (Clinicaltrials.Gov, 2020bo).

#### TJ003234

TJ003234, also known as TJM2 and TJ-003234RAR101, is an antigranulocyte macrophage–colony-stimulating factor (anti-GM-CSF) monoclonal antibody that produces a high level of mAbs against GM-CSF (Clinicaltrials.Gov, 2020bk). It is first discovered by a dynamic and global biotech company I-Mab Biopharma Co. Ltd. Again, this company arranged a multi-center, randomized, double-blind, placebo-controlled phase 1b/2 clinical trial (NCT04341116) to assess the safety and efficacy of its in COVID-19 (Clinicaltrials.Gov, 2020bl). In this study, 144 patients are assigned and divided into three groups to receive IV TJ003234 (3 mg/kg for 1^st^ group and 6 mg/kg for 2^nd^ group) and placebo (3^rd^ group).

#### Nivolumab

Nivolumab (Optivo^®^), a human IgG4 monoclonal antibody, is a programmed death 1 (PD-1)immune checkpoint inhibitor (ICI) that directly binds to the PD-1 ligand 1 (PD-L1) receptor and blocks it’s interaction capacity with the PD-L1 and -L2 (Wolchok et al., 2013). It is an approved drug, reversing T-cell anergy and boosting immune responses in several cancers, including metastatic melanoma, skin and lung cancer, and virus-associated tumors (CheckMate 358) and against the viral infections, including HIV (Le Garff et al., 2017; Topalian et al., 2017). To assess the safety, efficacy, and tolerability of intravenous nivolumab, a total of 92 patients with severe SARS-CoV-2 disease are randomly assigned in a randomized, multi-center, 2 parallel arms, open-label phase 2 clinical study and allocated in a 1:1 ratio for which they receive either nivolumab at a dose of 3 mg/kg on day 1 or standard care (Clinicaltrials.Gov, 2020bu).

#### Meplazumab

It is a humanized mAb that acts against host-cell-expressed CD147. Meplazumab blocks the infection of SARS-CoV-2 through binding with the S protein of SARS-CoV-2 (Bian et al., 2020). In a recent study, 20 COVID-19 patients with pneumonia are assigned in a single center, single-arm, open-label phase 2 clinical trial to receive meplazumab at an IV dose of 10 mg at 1^st^, 2^nd^, and 5^th^ day to evaluate the therapeutic safety, efficacy, and tolerability of this drug in COVID-19 (Bian et al., 2020).

#### Eculizumab

Eculizumab (Soliris), another approved humanized mAb for the inhibition of intravascular hemolysis of PNH, is a potent terminal complement inhibitor that directly binds to the C5 complement protein and inhibits the cleavage of C5a and C5b-9 (Legendre et al., 2013). In order to evaluate the safety and efficacy of this drug in COVID-19, the researchers of the Assistance Publique - Hôpitaux de Paris conducted a cohort multiple randomized controlled phase 2 trial (NCT04346797) where 120 patients with moderate or severe pneumonia were allocated to receive either eculizumab at a dose of 1,200 mg on days 1^st^, 4^th^, 8^th^ then 1,200 mg or 900 mg on day 12^th^ or best standard of care (Clinicaltrials.Gov, 2020w).

#### Clazakizumab

Clazakizumab is an anti-IL-6 monoclonal, which is a hereditarily engineered high affinity humanized monoclonal antibody (IgG1) that directly binds to IL-6 and averts its interaction and signaling through IL-6R (Eskandary et al., 2019). To determine the sustainable rate of virological response of this drug in COVID-19, patients with COVID-19 with signs of pulmonary involvement are randomly conducted in a phase 2, randomized, placebo-controlled intervention (NCT04348500) clazakizumab at the dose of 25 mg in 50 cc NS has been given by IV infusion x 1 dose and placebo at the dose of 50 cc NS given by IV infusion x 1 dose (Clinicaltrials.Gov, 2020i).

#### Avdoralimab

Avdoralimab (IPH5401), an immunoglobulin G1-kappa, is a selective anti-C5aR antibody that potentially reduces the inflammatory response in the lungs (World Health Organization, 2019). In response to current viral infection, 108 COVID-19 patients with severe pneumonia are included in a randomized, double-blind, placebo-controlled phase 2 clinical study (NCT04371367) to receive IV avdoralimab and placebo to improve the proportion of infected patients (Clinicaltrials.Gov, 2020e).

#### Lenzilumab

The humaneered recombinant antihuman granulocyte-macrophage colony- stimulating factor (anti-hGM-CSF) antibody lenzilumabis an IgG1 kappa monoclonal antibody that targets human GM-CSF to treat chronic myelomonocytic leukemia (Patnaik et al., 2019). To appraise the supportable rate of safety, efficacy and tolerability of virological response of it in COVID-19, a phase 3 randomized, double-blind, placebo-controlled intervention (NCT04351152) involving 238 patients with pneumonia are randomized in a 1:1 ratio of this drug plus standard care vs. standard care (Clinicaltrials.Gov, 2020ay).

#### LY3127804

LY3127804, a selective mAb firstly developed by an American pharmaceutical company Eli Lilly and Company, is engineered high affinity humanized monoclonal antibody (IgG4 isotype) that discerningly targets to angiopoietin-2 (Ang-2) and counteracts phospho-Tie2, tumor growth and metastasis (Chintharlapalli et al., 2016; Pestana et al., 2018). Eli Lilly and Company initiates a randomized, double-blind, placebo-controlled, phase 2 intervention (NCT04342897) of LY3127804 in April 13, 2020, to evaluate the effectiveness of LY3127804 against ongoing viral infection. In this study, 200 hospitalized patients with COVID-19 pneumonia have been receipted IV LY3127804 or placebo (Clinicaltrials.Gov, 2020bj).

#### IFX-1

It is applied as a first-in-class monoclonal antibody that serves as a C5a antagonist, which is being developed for the advanced treatment in COVID-19 patients by a German biopharmaceutical firm InflaRx in collaboration with Beijing (Clinicaltrialsarena.Com, 2020a). IFX-1 is one of the currently under development drugs generally used for the treatment as a skin disorder therapy, antiviral, antiinfective, antiinflammatory, and vascular disorder therapy. For the better advancement of this drug against COVID-19, the InflaRx has been assigned 130 COVID-19 patients with severe pneumonia in a two-arm (arm A: best supportive care plus IFX-1; arm B: best supportive care alone), randomized, open-label, pragmatic, adaptive, phase 2/3 clinical trial (NCT04333420) (Clinicaltrials.Gov, 2020aw).

#### Gimsilumab

A fully mAb gimsilumab that is developed by a pharmaceutical company Roivant Sciences Ltd. as a selective inhibitor of granulocyte-macrophage colony-stimulating factor (GM-CSF) (Clinicaltrialsarena.Com, 2020b). In case of COVID-19, the Roivant Sciences Ltd. has been included 270COVID-19 participants having ARDS and lung complication secondary in a phase 2, adaptive, randomized, double-blind, placebo-controlled, multi-center study (NCT04351243) to check its safety and efficacy. In this study, subjects receive either gimsilumab at a higher dose on day 1 and a low dose on day 8 or saline solution as placebo on day 1 and day 8 (Clinicaltrials.Gov, 2020bn).

#### Tocilizumab

A humanized mAb tocilizumab (Actemra^®^) is an FDA approved IL-6 inhibitor that selectively inhibits IL-6-mediated proinflammatory signaling by blocking both soluble and membrane-expressed IL-6 receptors (Schiff et al., 2011) and also interrupts the process of cytokine release syndrome (CRS) (Le et al., 2018). Additionally, it has already been approved for the treatment of several types of arthritis, including rheumatoid, polyarticular juvenile idiopathic, systematic juvenile idiopathic and polyarticular juvenile idiopathic arthritis (Oldfield et al., 2009; Le et al., 2018). In response to the ongoing pandemic COVID-19, the University Hospital Inselspital, Berneinitiates is conducting a randomized, multicenter, double-blind, placebo-controlled phase 2 clinical trial(NCT04335071) in collaboration with the Roche Pharma to check its safety and efficacy at a dose of 8 mg/kg body weight, with a maximum single dose 800 mg patients (n=100) with severe pneumonia compared to a placebo group (Clinicaltrials.Gov, 2020br).

#### Sarilumab

The first fully human mAb sarilumab (Kevzara^®^, REGN88, and SAR153191) developed by jointly Sanofi and Regeneron Pharmaceuticals is an inhibitor of IL-6Rα that is firstly approved for the treatment of rheumatoid arthritis (Sieper et al., 2015). Sarilumab has a potent ability to bind directly to the soluble and membrane‐bound IL‐6R with the selective affinity, in that way preventing IL‐6–mediated *cis* and *trans-signal*ing, thus may inhibit overactive inflammatory immune response associated with COVID-19 by inhibiting IL-6-mediated signaling (Genovese et al., 2015). Based on its IL-6 inhibitory capacity, the Regeneron Pharmaceuticals in collaboration with Sanofi starts have been started an adaptive phase 2/3, randomized, double-blind, placebo-controlled study (NCT04315298) to check out its clinical safety and efficacy in comparison to the control arm (Arrytown and Paris, 2020). For this, 2500 COVID-19 hospitalized patients with severe and critical phase are randomized to IV placebo or sarilumab at a single dose.

#### Bevacizumab

It is an anti-VEGF monoclonal IgG1 antibody that inhibits viral proliferation, migration, and survival by producing high levels of IgG1 antibody (Kazazi-Hyseni et al., 2010). In a randomized, open-label, controlled phase 2 clinical trial (NCT04344782), a most extensive hospital system in Europe (Assistance Publique - Hôpitaux de Paris) randomly assigns 130 patients with COVID-19 infection to receive either bevacizumab (7.5 mg/kg in 100 ml saline) as the experimental arm or standard of care as the control arm to examine safety and efficacy of this drug in COVID-19 (Clinicaltrials.Gov, 2020bt).

### Hormonal Preparations and Related Drugs

#### Aviptadil

Aviptadil, an injectable formulation of the vasoactive intestinal peptide (VIP), is usually used in PAH (Al-Saikhan et al., 2015). It has also been awarded by FDA Orphan Drug Designation for the ARDS treatment and admitted to the FDA CoronaVirus Technology Accelerator Program. Some nonclinical studies reported that the aviptadil selectively prevents N-methyl-D-aspartate (NMDA)-induced caspase-3 activation in lung and constrains the production of IL-6 and TNF-α (Clinicaltrials.Gov, 2020ao). In 20 year history, aviptadil shows the safety and efficacy for sarcoid, pulmonary fibrosis, bronchospasm, and erectile dysfunction in phase 2 trials and ARDS in phase 1 trial. For further assuring the safety and efficacy of this drug against ARDS, the multi-national company NeuroRx, Inc. initiates a randomized, placebo-controlled, multicenter phase 2 clinical trial (NCT04311697) in hospitalized patients (n = 120) with COVID-19 associated ARDS (Clinicaltrials.Gov, 2020ao). In this study, patients are assigned randomly to receive an IV infusion of aviptadil (50–150 pmol/kg/h over 12 h) plus maximal intensive care or standard saline infusion plus maximal intensive care.

#### Progesterone

The steroid hormone progesterone has traditionally been considered as the mammalian pregnancy hormone, which also reduces inflammation and promotes repair of the respiratory epithelium (Lydon et al., 1995; Hall and Klein, 2017). To evaluate safety and efficacy of progesterone against SARS-CoV-2, a phase 1 randomized, single center, controlled trial (NCT04365127) is conducted in which 40 patients (men) with COVID-19 who are 18 years of age or older receive either subcutaneous (SC) progesterone (100 mg/BID) plus standard care or standard care alone (Clinicaltrials.Gov, 2020bc).

#### Sildenafil

Sildenafil is an orally administered phosphodiesterase type 5 (PDE5) inhibitor that permits corpus cavernosum smooth muscle to relax and potentiating erections during sexual stimulation (Langtry and Markham, 1999; Georgiadis et al., 2020; Iordache et al., 2020a). It is also reported that the sildenafil can inhibit the breakdown of cyclic guanosine monophosphate (cGMP) through binding at the phosphodiesterase binding site (Iordache et al., 2020b; Rogosnitzky et al., 2020). A pilot study of sildenafil is designed in phase 3 clinical trial (NCT04304313) to check its citrate form tablet’s safety, efficacy, and tolerability at a dose of 0.1g/day for 14 days in 10 COVID-19 patients (Clinicaltrials.Gov, 2020ba).

#### Triiodothyronine (T3)

It is a thyroid hormone that usually impedes the activation of p38 mitogen-activated protein kinase (MAPK) and promotes tissue repair through controlled protein kinase B (Akt) activation (Pantos et al., 2020). To evaluate its anti-COVID-19 activity, a phase 2, parallel, prospective, randomized, double-blind, placebo-controlled trial (NCT04348513) is designed to explore the probable effect of IVT3 solution (0.8 g/kg within 1 h and then followed by 0.113 g/kg/h for 48 h) in critically ill patients admitted in the intensive care unit (ICU) due to COVID-19 (Clinicaltrials.Gov, 2020bw).

#### Estradiol Patch

It is a nuclear hormone that interacts with a target cell receptor (Erα or Erβ) within the cytoplasm of the cell. The *in vivo* and *in vitro* studies have been demonstrated that estrogen acts in different types of viral infections and wound repair processes (Clinicaltrials.Gov, 2020aj). Thus, the it can be used in viral infections in the lung. To reduce the severity of SARS-CoV-19 disease, COVID-19 positive and probable COVID-19 positive patients (n = 110) are randomly assigned to receive estradiol patchat the dose of 100 µg/day for 7 days on the skin (Clinicaltrials.Gov, 2020aj).

### Cardiovascular Drugs

#### Losartan

Losartan, a selective, orally available ACE inhibitor, which was developed to treat heart failure that acts through blocking the vasoconstrictor and aldosterone-secreting effects of angiotensin II *via* inhibiting the binding of it to the angiotensin II type 1 receptor (AT1R) (Tsatsakis et al., 2019; Tignanelli et al., 2020). Around 14% dose of losartan is converted to its 10 to 40 fold more active metabolite E3174 after an oral administration of it with its 6 to 9 h estimated terminal half-life. A research group led by the University of Minnesota has recently initiated a randomized, placebo-controlled, multi-center, double-blinded, phase 2 study (NCT04312009) for COVID-19 treatment (Clinicaltrials.Gov, 2020au). In this study, investigators assigned 200COVID-19 patients in a 1:1 ratio to get this drug at an oral dose of 50 mg/day or placebo for 7 days or hospital discharge (Clinicaltrials.Gov, 2020au).

#### Valsartan

A highly selective angiotensin II (Ang II) type 1 (AT_1_) receptor blockers that potentially increases pulmonary vascular permeability through blocking AT1R activation and down-regulating the activity ofACE2 (Markham and Goa, 1997). It is evident that the COVID-19 is a high burden of morbidity and mortality because of the development of ARDS. The renin-angiotensin-system (RAS) is also related to developing ARDS and in the meantime, ACE2 is one of the enzymes involved in the RAS cascade (Trifirò et al., 2020). According to this perspective, some scientists of Radboud University initiate a double-blind, placebo-controlled 1:1 randomized phase 4 intervention (NCT04335786) in a total of 651 COVID-19 patients to treat them with valsartan in a dosage titrated to blood pressure up to a maximum of 160 mg/BID or placebo (80 or 160 mg) for 14 days or hospital discharge (Clinicaltrials.Gov, 2020bx).

#### Ramipril

Oral capsule ramipril is suggested as another RAS blocker that averts diabetes in people with hypertension or cardiovascular disease (Bosch et al., 2006). Exhibiting comparable pharmacodynamic responses to captopril and enalapril, ramipril is also considered as a long-acting ACE inhibitor (Todd and Benfield, 1990). It is a prodrug that is converted to its pharmacologically active metabolite ramiprilat after absorption through hydrolysis with a long estimated terminal half-life (Todd and Benfield, 1990). In response to ongoing infectious disease, the University of California, San Diego in collaboration with Pfizer is planning to initiate a randomized, double-blind, placebo-controlled, phase 2 trial (NCT04366050) to treat 560 COVID-19 patients with ramipril (2.5 mg/day) or placebo for 14 days (Clinicaltrials.Gov, 2020bd).

#### APN01

It is a recombinant humanACE2 (rhACE2), which is currently used for COVID-19 patients due to its ability to block viral entry and decreasing viral replication in the host cells (Taylor, 2020). APN01 is being tried to develop by the Apeiron Biologics for advanced treatment of COVId-19. For this reason, recently, a randomized, double-blind, phase 2 trial (NCT04335136) of APN01 is assigned in 200 COVID-19 participants (Clinicaltrials.Gov, 2020bg).

#### Spironolactone

It is an antagonist of aldosterone often used to treat patients with low renin essential hypertension, primary aldosteronism, hypokalemia, and diuretic (Loriaux et al., 1976). It acts as a competitive aldosterone antagonist through binding at the aldosterone-dependent Na^1+^/K^1+^ exchange site in the distal convoluted renal tubule. A phase 4 clinical study (NCT04345887) is designed by Istanbul University-Cerrahpasa to assess the effects of spironolactone on oxygenation in COVID-19 ARDS patients (Clinicaltrials.Gov, 2020bi).

### Agents Acting on Blood and Blood-Forming Organs

#### Nafamostat Mesilate

It is a proven serine protease inhibitor that is initially approved in Japan for the treatment of disseminated intravascular coagulation, acute pancreatitis, and anticoagulation in extracorporeal circulation (Tu et al., 2020). It inhibits different types of enzymatic systems, including coagulation and fibrinolytic systems (e.g., thrombin, Xa, XIIa), complement system, and kallikrein-kinin system (Bittmann et al., 2020). It has also been recognized that the nafamostat mesilate is an inhibitor of MERS-CoV S glycoprotein mediated viral membrane fusion through the inhibition of transmembrane protease, serine 2 (TMPRSS2) activity (Yamamoto et al., 2016). Based on previous experiments, a randomized, double-blind, placebo-controlled parallel-group, phase 2/3 trial (NCT04352400) of nafamostat mesilate is designed to evaluate its efficacy against 256 COVID-19 patients (Clinicaltrials.Gov, 2020ai).

#### Camostat Mesilate

It is another synthetic serine protease inhibitor that is initially used to treat dystrophic epidermolysis, chronic pancreatitis, and oral squamous cell carcinoma (Tu et al., 2020). It was first manufactured by the Nichi-Iko Pharmaceutical Co., Ltd. in combination with Ono Pharmaceutical, Japan (Ohkoshi and Oka, 1984). It has experimented that the camostat mesilate showed the inhibition effect of SARS-COV-2 replication in the *in vitro* study. Based on this previous preclinical study, a phase 2 clinical study is designed with 114 COVID-19 patients to treat them either with camostat mesylate at a dose of 200mg/TID or with placebo/TID for 7 days (Clinicaltrials.Gov, 2020g).

### Vitamins

#### Vitamin C

Vitamin C (also known as L-ascorbic acid, ascorbic acid, and ascor, sodium ascorbate), a six-carbon lactone, is popular for its antioxidant properties that plays an essential role in reducing the inflammatory process, preventing from respiratory failure, deterring common cold, inhibiting the neutrophils accumulation in the lung, and also modulating the immune system (May and Harrison, 2013; Wilson, 2013; Carr and Maggini, 2017; Salehi et al., 2019b; Sharifi-Rad M. et al., 2020). In addition, some previous studies have been highlighted that the higher dose of IV vitamin C may be beneficial for the patients with acute lung injury, ARDS, and sepsis (Salehi et al., 2019a; Clinicaltrials.Gov, 2020c). It has also been reported that the deficiency of this vitamin may increase the risk and severity of influenza infections (Clinicaltrials.Gov, 2020by). Based on the previous reports, some clinical trials on this vitamin have been registered in order to evaluate its effectiveness in COVID-19.

A research group led by the Hunter Holmes Mcguire Veteran Affairs Medical Center has registered a nonrandomized, open-label, parallel, phase 1/2 clinical trial (NCT04357782) to evaluate the safety, tolerability, and efficacy of the IV vitamin C against SARS-CoV-2 infection and decreased oxygenation (Clinicaltrials.Gov, 2020c). In response to this study, 20 hospitalized patients (age: 18-99 years) are designed to receive this vitamin at a dose of 50 mg/kg given 6 hourly for 4 days (16 total doses) (Clinicaltrials.Gov, 2020c). Another research team of Zhongnan Hospital is actively functioning on vitamin C to evaluate its therapeutic efficacy against the severe SARS-CoV-2 infected pneumonia patients (n = 140) of 18 years and older-aged humans (Clinicaltrials.Gov, 2020by). This study is assigned in a randomized, placebo-controlled, phase 2 clinical trial (NCT04264533) to treat the patients with either vitamin C (12 g/BID for 7 days) plus sterile water (50 ml) or sterile water alone (50 ml/BID for 7 days) (Clinicaltrials.Gov, 2020by). In addition, vitamin C is also introduced in another randomized, multi-center phase 2 (NCT04395768) intervention in 200 COVID-19 patients (18 years and older), which is led by the National Institute of Integrative Medicine, Australia (Clinicaltrials.Gov, 2020an). In this study, the recommended dose of vitamin C is 50 mg/kg 6 hourly on day 1 followed by 100 mg/kg 6 hourly for 7 days (Clinicaltrials.Gov, 2020an).

#### Vitamin D

Vitamin D may provide the boosting and priming effects against the viral replication caused by several microbial peptides including cathelicidins and defensins (Grant et al., 2020), dysregulation of the renin-angiotensin system, and cytokine storm in the host (Clinicaltrials.Gov, 2020x) through modulating the innate and adaptive immune system (Aranow, 2011). According to the various pre-clinical studies, it is found that the SARS-CoV-2 replication in the host cell leads to severe ARDS by leading to a cytokine storm (Clinicaltrials.Gov, 2020x). Meanwhile, various studies (*in vivo* and *in vitro*) on vitamin D have been established that clearly highlight the activity of vitamin D against ARDS and COVID-19-associated coagulopathy (Clinicaltrials.Gov, 2020aq; Clinicaltrials.Gov, 2020x). Based on this critical information on vitamin D, some research groups are continuously working on clinical trials of vitamin D to evaluate its response against COVID-19 patients.

Recently, the University Hospital, Angers has registered a multicenter, randomized, phase 3 clinical trial (NCT04344041) to check out the efficacy of vitamin D for COVID-19 patients (Clinicaltrials.Gov, 2020x). In this instance, 260 patients with life-threatening COVID-19 are randomly allocated to get a high dose of oral vitamin D3 (400,000 IU) or a standard dose of vitamin D3 (50,000 IU) (Clinicaltrials.Gov, 2020x). Another institution Louisiana State University Health Sciences Center in New Orleans has also decided to initiate a multi-center, prospective, randomized, phase 2 intervention (NCT04363840) on SARS-CoV-2 infected patients (n = 1,080) to appraise the efficiency of the vitamin D (50,000 IU, once weekly for 2 weeks) in combination with aspirin (81 mg, once daily for 14 days) against the growing global health crisis (Clinicaltrials.Gov, 2020aq).

At the current crisis of COVID-19 pandemic, the investigators from the Hospital de Alta Complejidad en Red El Cruce Florencio Varela, Buenos Aires, Argentina design a phase 4, randomized, placebo-controlled clinical trial (NCT04411446) of vitamin D in 1,265 hospitalized COVID-19 patients to identify the outcome of vitamin D at a dose of 100.000 UI (total five capsules) compared with placebo at the similar dosage of vitamin D (Clinicaltrials.Gov, 2020h). Besides, to determine the therapeutic efficiency of vitamin D3, a randomized, double-blind, placebo-controlled, proof-of-concept, phase 2 intervention (NCT04400890) has been registered. In response to this study, 200 participants are randomly apportioned in 2 arms (100 for active comparator and 100 for placebo comparator) to treat them with either resveratrol plus vitamin D3 (100,000 IU on day 1) or placebo with vitamin D3 (100,000 IU on day 1) for 15 days (Clinicaltrials.Gov, 2020be).

## Convalescent Plasma Therapy

**Figure 3:**
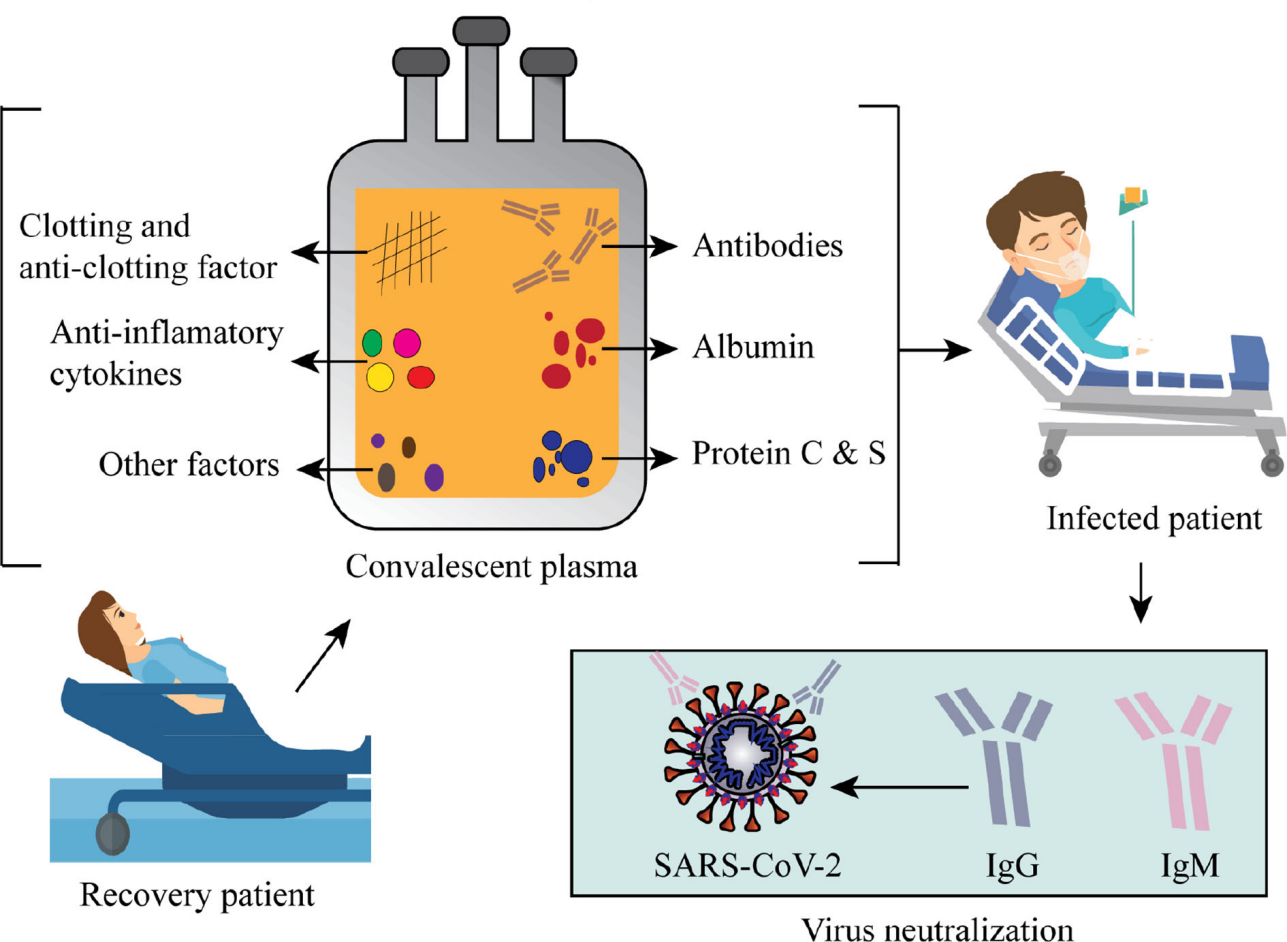
Schematic represents convalescent plasma components and the viral neutralizing activity of convalescent plasma.

Passive immunotherapy is one of the effective therapeutic approaches in the endemic or pandemic infectious disease, which is still used in ongoing pandemic expending polyclonal antibodies, rather as a hyperimmune preparation from the convalescent patient’s sera who have already recovered from the infection (Dodd, 2012). Convalescent sera or immunoglobulin obtained from the donor is very effective in SARS-CoV-2 infected patients by emerging immediate immune responses in the host system will be possible to neutralize the viral particles in the host (**Figure 3**) (Rojas et al., 2020).

Passive immunotherapy has been used as a reliable treatment option for many infectious outbreaks, including the 2003 SARS-CoV-1 epidemic, 2009-2010 H1N1 influenza virus pandemic, 2012 MERS-CoV epidemic, and 2014 Ebola virus epidemic (Chen L. et al., 2020). Based on the previous experiences of CPT in viral infections, the clinical trials of convalescent plasma (CP) in different countries have been assigned to evaluate the safety, efficacy, and immunogenicity of passive immunotherapy for the treatment of COVID-19. As of now on 9^th^ June 2020, approximately 40 clinical studies on the CP have been registered with the clinicaltrial.gov for SARS-Cov-19 infection. Based upon inclusion and exclusion criteria, we select a total of 15 clinical trials that are registered from May 2020 to Jun 2020 (**Table 2**).

**Table 2:** Convalescent plasma therapy in clinical studies.

Registry number	Sponsor	No. of patients (Age)	Dose/conc.	Phase (Status)	References
NCT04377568	The Hospital for Sick Children	100(up to 18 years)	10 ml/kg	2(Not yet recruiting)	(Clinicaltrials.Gov, 2020ag)
NCT04389944	University Hospital, Basel, Switzerland	15(18 years and older)	200 ml	Not applicable(Recruiting)	(Clinicaltrials.Gov, 2020d)
NCT04390178	Joakim Dillner	10(18–80 years)	180–200 ml	1 and 2(Active, not recruiting)	(Clinicaltrials.Gov, 2020o)
NCT04384497	Joakim Dillner	50(18 years and older)	200 ml	1 and 2(Recruiting)	(Clinicaltrials.Gov, 2020t)
NCT04389710	Thomas Jefferson University	100(18 years and older)	200–600 ml	2(Recruiting)	(Clinicaltrials.Gov, 2020q)
NCT04407208	Biofarma	10(18 years and older)	Three times of each 100 ml	1(Recruiting)	(Clinicaltrials.Gov, 2020u)
NCT04372979	Direction Centrale du Service de Santé des Armées	80(18–80 years)	Two units of units of each 200–230 ml	3(Not yet recruiting)	(Clinicaltrials.Gov, 2020af)
NCT04380935	Indonesia University	60(18 years and older)	Not given	2 and 3(Not yet recruiting)	(Clinicaltrials.Gov, 2020z)
NCT04403477	Bangabandhu Sheikh Mujib Medical University, Dhaka, Bangladesh	20(16 years and older)	200 and 400 ml	2(Recruiting)	(Clinicaltrials.Gov, 2020v)
NCT04408209	National and Kapodistrian University of Athens	60(18 years and older)	Not given	Not applicable(Recruiting)	(Clinicaltrials.Gov, 2020r)
NCT04383535	Hospital Italiano de Buenos Aires	333(18 years and older)	5–10 ml/kg/h	Not applicable(Not yet recruiting)	(Clinicaltrials.Gov, 2020n)
NCT04391101	Hospital San Vicente Fundación	231(18 years and older)	400 and 500 ml	3(Not yet recruiting)	(Clinicaltrials.Gov, 2020s)
NCT04395170	Lifefactors Zona Franca, SAS	75(18 years and older)	200–250 ml	2 and 3(Not yet recruiting)	(Clinicaltrials.Gov, 2020p)
NCT04374149	Prisma Health-Upstate	20(12–80 years)	Not given	2(Not yet recruiting)	(Clinicaltrials.Gov, 2020bq)
NCT04383548	Assiut University	100(21–50 years)	Not given	Not applicable(Not yet recruiting)	(Clinicaltrials.Gov, 2020j)

A research team led by the Hospital for Sick Children in Canada registered a multi-centered, open-label, randomized controlled phase 2 clinical trial (NCT04377568) to evaluate the efficacy and safety of COVID-19 CP (C19-CP) for the treatment of COVID-19 in hospitalized children (Clinicaltrials.Gov, 2020ag). In this study, 100 hospitalized children (age up to 18 years) are randomized (1:2 ratio) to receive either C19-CP at the dose of 10 ml/kg plus standard care or standard care. Another research team of the University Hospital, Basel, Switzerland is energetically functioning on a pathogen-inactivated CP addition to best supportive care and antiviral therapy on experimental worsening in participants (n = 15) of 18 years and older age with COVID-19 (Clinicaltrials.Gov, 2020d).

An open-label, nonrandomized, controlled, phase 1/2 clinical trial (NCT04390178) is being carried out by the Joakim Dillner to evaluate the efficacy, safety, and tolerability of plasma collected from the donors who have recovered from the SARS-Cov-19 infection. In response to this study, 10 participants with varying degrees of COVID-19 illness are assigned nonrandomly to receive 180–200 ml of CP (Clinicaltrials.Gov, 2020o). Another nonrandomized, phase 1/2 clinical trial (NCT04384497) is designed by Joakim Dillner with 50 participants (age: 18 years and older) to treat them with CP (200 ml, up to a maximum of 7 CP infusions) for further investigation (Clinicaltrials.Gov, 2020t).

The Thomas Jefferson University registered an open-label, phase 2 clinical intervention (NCT04389710) with 100 SARS-CoV-2 infected participants who have severe or life-threatening COVID-19. The participants typically receive 1–2 units (200–600 ml) of ABO compatible donor’s CP administrating at a rate of 100–250 ml/h, which has the anti-SARS-CoV-2 antibody (Clinicaltrials.Gov, 2020q).

Most recently, a pilot study led by the Biofarma has enrolled 10 participants(age: 18 years and older) with severe COVID-19 at Gatot Soebroto Central Army Presidential Hospital Jakarta Pusat, Indonesia has undergone with the CP administrating at the 3 times of each 100 ml on day 0, 3, and 6, which has the minimum titer (1:80) of anti-SARS-CoV-2 antibody (Clinicaltrials.Gov, 2020u). On the other hand, a phase 3 clinical study involved in PlasCoSSA (randomized, controlled, triple-blinded, parallel study) is registered to evaluate the efficacy of the transfusion of SARS-CoV-2 CP as an early treatment of the COVID-19 (Clinicaltrials.Gov, 2020af). In this instance, 80 participants of 18-80 years aged are randomly conducted to receive an amotosalen inactivated IV injection of 2 units SARS-CoV-2 CPof each 200–230 ml (Clinicaltrials.Gov, 2020af).

Recently, the Indonesia University has registered for initiating a phase 2/3, randomized, open-label, controlled clinical study (NCT04380935) in the Referral Hospitals in Indonesia to evaluate the effectiveness and safety of CPT in COVID-19 patients with ARDS (Clinicaltrials.Gov, 2020z). In response to this study, the research group of Indonesia University is planning to assign 60 patients randomly to get either CP plus standard care or standard care (Clinicaltrials.Gov, 2020z).

The Bangabandhu Sheikh Mujib Medical University, Dhaka, Bangladesh has recently registered a phase 2, randomized, three-arm clinical trial with 20 participants testing positive for SARS-CoV-2 (Clinicaltrials.Gov, 2020v). Of interest, the apheretic CP is collected from donors who have recovered from COVID-19, which has the antibody titre >1:320. In this study, the intervention model is designed as the three arms (arm-A, B, and C) in which the participants are conducted to receive standard supportive treatment alone as the arm-A, standard supportive treatment plus 200 ml apheretic CP as the arm-B, and standard supportive treatment plus 400 ml apheretic CP as the arm-C to evaluate effectiveness, safety, and efficacy of the dose-depended CPT (Clinicaltrials.Gov, 2020v).

In addition, to determine the therapeutic efficacy of the CP, the titer of neutralizing anti-SARS-CoV-2 antibodies (IgG) obtained from the CP of the fully recovered patients from COVID-19 is administered on days 1–7, 14, 21, 28, and 35 from the start of treatment in 60 patients (age: 18 years and older) with severe SARS-Cov-19 infection (Clinicaltrials.Gov, 2020r). Another multi-center randomized, double-blind, placebo-controlled clinical trial (NCT04383535)has been also registered by the Hospital Italiano de Buenos Aires to evaluate the effect of CP vs. placebo (Clinicaltrials.Gov, 2020n). For this study, 333 patients with severe COVID-19 are conducted in a 2:1 ratio, to administer CP (222 patients) or placebo (111 patients). On the other hand, a phase 3 clinical study (NCT04391101) with 231 participants in a 2:1 ratio (CP:standard management), registered from Hospital San Vicente Fundación is running to evaluate the safety, efficacy, and tolerability of CP (400–500 ml) (Clinicaltrials.Gov, 2020s).

At the current crisis of the nCoV-19 pandemic, the investigators from Lifefactors Zona Franca, SAS designs a randomized, multicenter, phase 2/3 clinical trial (NCT04395170) of CP in 75 hospitalized patients with COVID-19 to assess the efficacy of CP at a dose of 200–250 ml on days 1 and 3 of the intervention compared to the intravenous anti-COVID-19 human immunoglobulin at a dose of immunoglobulin 10% IgG solution on days 1 and 3 of treatment (Clinicaltrials.Gov, 2020p).

Therapeutic plasma exchange (TPE) is an important intervention that helps instantly and scientifically to remove pathogenic antibodies and toxic candidates by using centrifugal separation of plasma or plasma membrane filtration. Sometimes, TPE in combination with tocilizumab and steroids has been used efficaciously for the treatment of severe 2, 3, 4 CRS following CAR-T treatment. To evaluate the efficacy of TPE, the Prisma Health-Upstate has registered a pilot study, where 20 patients are enrolled in a nonrandomized, open-label phase 2 clinical trial receive either TPE alone and or in combination with ruxolitinib (Clinicaltrials.Gov, 2020bq). In another study, the hyper immunoglobulins containing anti-SARS-CoV-2 immunoglobulin is being investigated in order to assess its efficacy as a passive immunization as well as treatment of early disease before the development of lower respiratory tract disease (e.g., pneumonia) (Clinicaltrials.Gov, 2020j).

## Discussion: Challenges and Clinical Perspectives on COVID-19 Pharmacotherapy

It is the greatest challengeable for the rapid identification of effective therapy developmental technologies and interventions for the COVID-19 associated paramount global public health crisis.

**Figure 4:**
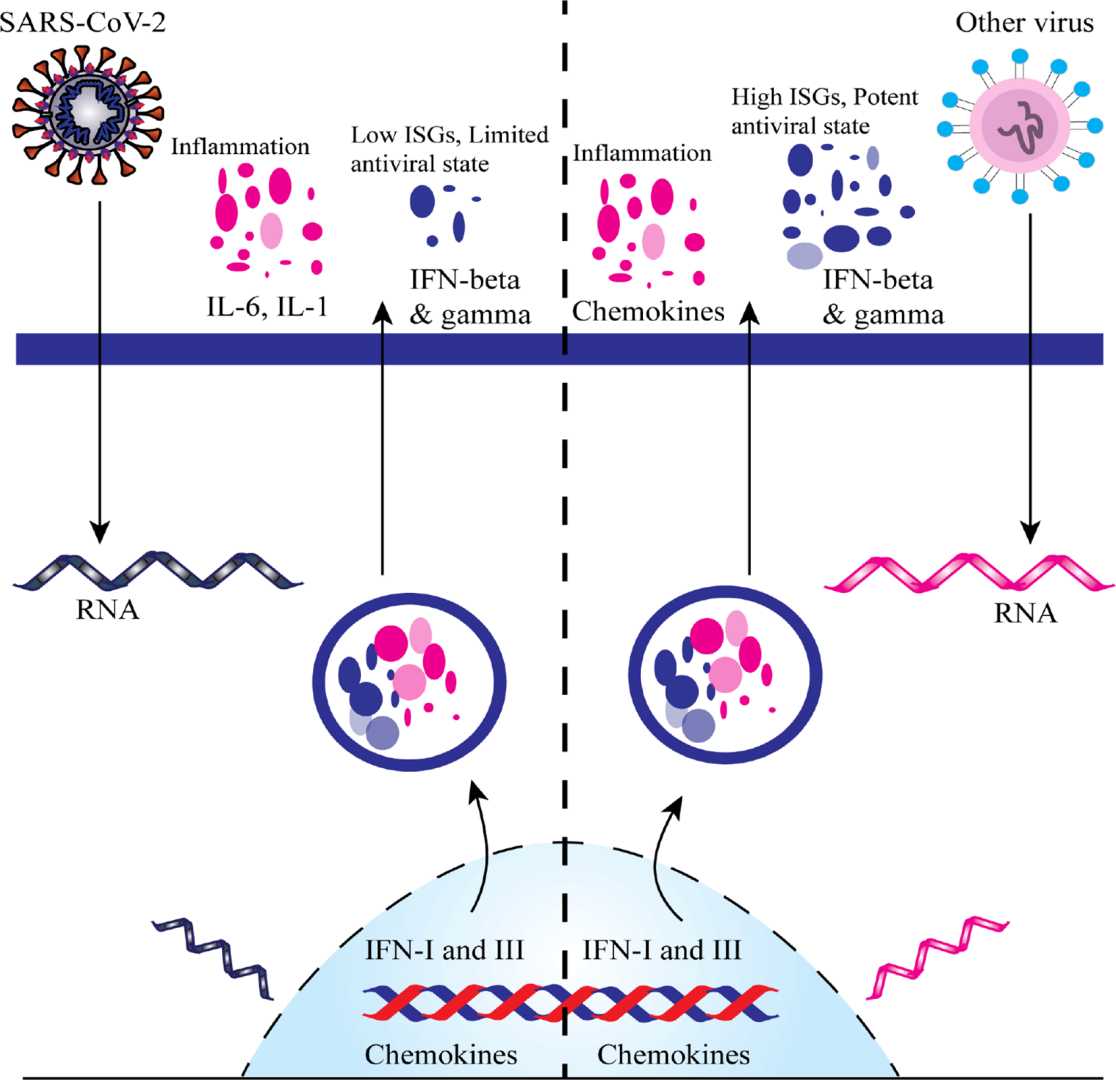
A comparative scheme regarding the imbalanced host response of Severe Acute Respiratory Syndrome Coronavirus 2 (SARS-CoV-2) infection versus other common respiratory virus infections. ISGs, IFN-stimulated genes.

Compared with other viral infection, SARS-CoV-2 causes high proinflammatory disease state associated with COVID-19 through inducing lower levels of IFN–I and –III expression with a moderate reaction of IFN-stimulated genes (ISGs) and raising chemokine expression (**Figure 4**) (Blanco-Melo et al., 2020).

Mild forms of COVID-19 can be treated at home if the infection is not very symptomatic and the person can be properly isolated (Godman, 2020). Patient care in these cases focuses on preventing transmission to others and monitoring the clinical condition to detect damage that could lead to hospitalization (Gao et al., 2020). Patient care in these cases is purely symptomatic (antipyretic), and preventive - the use of a mask in contact with other people, surface disinfection, hand hygiene, isolation of other people (Gao et al., 2020).

In other more severe clinical forms of COVID-19, patient care consists of the following aspects (Wiersinga et al., 2020):

- Initially, symptomatic treatment is used - antipyretics to control fever (Jamerson and Haryadi, 2020)
- In case of hypoxia - oxygen therapy to maintain saturation> 94% - in patients who have signs of aggravation (apnea or severe dyspnea, central cyanosis, shock, coma, convulsions) - airway management, oxygen therapy minimum 5 L/min up to 10–15 L/min per mask; after stabilization SpO2 (blood oxygen saturation levels)> 90% is maintained; in some cases noninvasive ventilation is recommended (Dondorp et al., 2020).
- Co-infection treatment - even in case of suspicion of COVID-19, empirical antibiotic therapy is administered, especially in case of sepsis (1 h after the identification of sepsis), based on the clinical diagnosis (Chang and Chan, 2020).
- In case of ARDS (acute respiratory distress syndrome) - mechanical ventilation; with extreme care during the intubation maneuver which has a high risk of contamination; in severe ARDS it is recommended to use the sitting position; hydroelectrolytic rebalancing
- Septic shock - hydroelectrolytic rebalancing antibiotic in the first hour, installation of central venous/arterial catheter (Fan et al., 2020).
- Prevention of complications is essential - maneuvers specific to each type of complication, especially in the case of those determined by prolonged immobilization and parenteral nutrition(Phua et al., 2020);
- The next supportive medicines will not be administered (due to the fact that they can aggravate the patient’s condition) - hypotonic crystalloids, corticosteroids (no benefits have been proven so far, but many side effects and increased mortality due to secondary infections and side effects, to be used only if there are comorbidities that would may require such therapy) (Singh et al., 2020).

The reason for using corticosteroids in COVID-19 therapy is based on their ability to reduce the host’s inflammatory responses in the lungs, inflammatory responses that could cause acute lung damage and acute respiratory distress syndrome. However, this benefit may be outweighed by their side effects, including delayed viral clearance and increased risk of secondary infection. Although direct evidence for the use of corticosteroids in COVID-19 is limited, analyzes of results in other viral pneumonias are relevant (Health, 2020a).

Observational studies in patients with SARS and MERS did not report any association between corticosteroid use and increased survival (Arabi et al., 2018), but showed an association of their use with delayed viral clearance in the respiratory tract and blood and the high frequency of complications, including hyperglycemia and psychosis (Keller et al., 2020).

In addition, a 2019 meta-analysis of 10 observational studies with 6,548 patients with influenza pneumonia found that corticosteroid therapy was associated with an increased risk of mortality (risk ratio [RR], 1.75 [95% CI, 1.3–2.4]; P <0.001) and a twice as high risk of secondary infections (RR, 1.98 [95% CI, 1.0–3.8]; P = 0.04)) (Ni et al., 2019).

Although the effectiveness of corticosteroids in acute respiratory distress syndrome and septic shock remains generally controversial, Russell and colleagues have argued that they are more effective in bacterial infections than in viral ones. A recent retrospective study of 201 COVID-19 patients in China found that for those who developed acute respiratory distress syndrome, methylprednisolone treatment was associated with a lower risk of death (23/50 [46%] with steroids vs. 21/34 [62%] without; HR, 0.38 [95% CI, 0.20–0.72]) (Russell et al., 2020).

Therefore, the potential adverse reactions and lack of proven benefits for corticosteroids in COVID-19 are arguments against their routine use in patients with COVID-19, unless there is a concomitant convincing indication, such as chronic exacerbation of obstructive disease or refractory shock.

Very recently, according to preliminary results from the RECOVERY study, dexamethasone, a common steroidal antiinflammatory drug, could reduce the death rate by one-third among patients severely affected by the new coronavirus. Dexamethasone is a glucocorticoid used since the 1960s in the treatment of many inflammatory conditions, but also in oncology (Bucolo et al., 2018).

Launched in March 2020, RECOVERY (Randomized Evaluation of COVid-19 Therapy) is one of the largest studies exploring potential treatments for COVID-19, and has included approximately 11,500 patients from over 175 hospitals NHS (National Health Service) in the United Kingdom. The results were surprising: one of the arms of the study, in which 2,104 patients were treated with six milligrams of dexamethasone per day (orally or intravenously) for ten days, was compared with 4,321 patients who received treatment considered be the current standard for SARS-CoV-2 infection (RECOVERY Collaborative Group, 2020).

In patients receiving standard treatment, mortality at 28 days was 41% in patients who required invasive ventilation, 25% in those who required only oxygen, and was lower (13%) in those who did not have need any intervention. It has been found that the use of dexamethasone reduces mortality by one third in ventilated patients - ratio 0.65; 95% confidence interval (0.48–0.88); p = 0.0003 - and one-fifth in patients with additional oxygen requirements - 0.80 (0.67–0.96); p = 0.0021, no significant benefit was observed in patients who did not require respiratory support - 1.22 (0.86 to 1.75); p = 0.14. These results demonstrate that if patients with COVID-19 requiring additional oxygen or invasive ventilation are given dexamethasone, it could save lives at extremely low cost. However, the WHO warns that the use of dexamethasone should only be used in severe cases of SARS-CoV-2 infection, these being the situations in which benefits were noticed significant (Villar et al., 2020).

Thus, dexamethasone should become the new standard in the treatment of COVID-19, especially in severe cases and the WHO will soon update the therapeutic protocol guidelines for COVID-19 (WHO, 2020).

In the case of drug development, the shorter time period insisted health providers to focus on identifying existing drugs or drug candidates intended for other indications that may have efficacy against COVID-19 and put them into accelerated clinical trials. Further dose assessments can be combined into an extended phase 3 trial using a combination of clinical, viral load decline and immune response as endpoints. This kind of accelerated procedure will place an extensive load on controlling agencies that only the pandemic itself can rationalize. To solve this, WHO launched the harmonized “Solidarity Trial” in different countries to rapidly assess in thousands of COVID-19 patients to evaluate the effectiveness of current antiviral and antiinflammatory agents not yet evaluated specifically for COVID-19 (Cheng et al., 2020). Similarly, The US National Institute of Allergy and Infectious Diseases (NIAID) commenced an adaptive design for international phase 3 trial called “ACTT” to include up to 800 hospitalized COVID-19 persons at 100 places in numerous countries (Clinicaltrials.Gov, 2020b).

However, the more international arrangement would necessitate maintaining the high degree of regulatory coordination and normalization of clinical operations across many diverse settings. Another aspect of regarding drug development in COVID-19 is repurposed drugs, an accepted drug for the treatment of different ailments or medical conditions than that for which it was initially developed. Conversely, COVID-19 is a novel disease, the repurposed drugs will not totally effective, and extensive research will be needed to optimize them (Shi et al., 2020).

Like the other two therapies, CPT was a promising treatment for serious COVID-19, though it has hidden risks such as aggravating hyperimmune attacks. Moreover, this therapy is more effective in the earlier stage of disease and researches on SARS confirmed it. Therefore, the ideal timing of administering CPon COVID-19 patient needs to be cautiously measured (Zhao and He, 2020). Another challenging factor of CPT is SARS-CoV-2 neutralizing antibody titer. An investigation on SARS verified that the precise IgG began to upsurge about week 3 later of the onset, and reached at a high level at week twelve (Li et al., 2003). In addition, another study on influenza advocated that CPT with a neutralizing antibody titer level of 1:160 and more reduced mortality. Thus, CP from donors who have recovered at week 12 after onset with a neutralizing antibody titer level of not less than 1:160 is estimated to be more effective (Hung et al., 2011). Besides, the most common adverse reaction of CPT are transfusion-related problems, including fever, anaphylactic shocks, transfusion-related acute lung injury, circulatory overload and hemolysis (Maclennan and Barbara, 2006). Considering all these challenges, healthcare providers may use CPT for hospitalized patients to reduce morbidity and mortality.

Herd immunity (HdI) is the indirect protection from infection conferred to susceptible individuals when a sufficiently large proportion of immune individuals exist in a population. The time to reach HdI of a community depends on the reproduction number (R_0_). It means the average number of people that a single infected person with the virus can infect those aren’t already immune. The higher the R_0_, the more people need to be resistant to reach HdI (Randolph and Barreiro, 2020). According to the scientific reports, the R_0_ for COVID-19 is within 2 to 6 (Sanche et al., 2020). This means that one infected person can infect two to six other persons. It also means 17 to 50% of the population would need to be resistant before HdI kicks in and the infection rates start to go down. However, a single pathogen may have multiple R_0_ values depending on the characteristics and transmission dynamics of the population. Therefore, the HdI threshold value (1 - 1/R_0_) may vary between populations (Anderson and May, 1985).

The communicability of an infectious disease depends on many factors, such as population density and age structure, cultural behaviors, underlying comorbidity rates, differences in contact rates across demographic groups, which may affect the HdI threshold (Sharifi-Rad J. et al., 2020). The effective reproduction number (R_e_ or R_t_) is also important to understand the population-level immunity. It is the average number of secondary cases generated by a single index case over an infectious period in a partially immune population. Thus, the goal of vaccination programs is to bring the value of R_e_ below 1 will be possible only when the HdI threshold exceeded. The pathogen spread cannot be maintained, therefore, a decline in the number of infected individuals will be seen within the population (Randolph and Barreiro, 2020).

The challenges of HdI in case of COVID-19 are: (i) less effectiveness, periodic outbreaks can still occur, (ii) unevenly distributed within a population, clusters of susceptible hosts that frequently contact one another may remain, (iii) the proportion of immunized individuals surpasses the HdI threshold, susceptible individuals will be found in the risk zone for local outbreaks, (iv) nonrelevant infection fatality rate (IFR) and case fatality rate (CFR). Still there is no straightforward, ethical path to reach the goal with HdI in case of COVID-19, due to the societal consequences of achieving it are devastating. A nonuniform COVID-19 case fatality rate (CFR) has been reported across age groups, with the vast majority of deaths occurring among individuals 60 years old or greater. Sex- and ethnicity-specific CFRs suggest that genetic, environmental, and social determinants may affect in susceptibility to COVID-19 and the severity of SARS-CoV-19 infections.

Sodium chloride (NaCl) also called ‘table salt’ as coating material on the fiber surface of the filtration unit of surgical mask effectively deactivated a number influenza virus species, suggesting a new strategy in the protective measures to avoid primary/secondary infection and transmission of many viruses, including SARS-CoV-19 (Quan et al., 2017). On the other hand, the natural adsorbents, including clay, charcoal, and clay minerals showed 99.99% adsorption of CoVs (Robson, 2020). Some minerals that may act against CoVs are selenium (Ma et al., 2019), copper (Rupp et al., 2017), iron (Jayaweera et al., 2019), chromium (Terpiłowska and Siwicki, 2017), potassium (Punch et al., 2018), zinc (Te Velthuis et al., 2010), and so on. Moreover, medicinal plants or their derivatives are also evident to act against hCoVs (Kim et al., 2010; Aanouz et al., 2020; Islam et al., 2020).

## Limitations

The main limiting aspects of this comprehensive review emerge from the studies that have been performed on these drugs that are still only experimental. Many of the studies analyzed included a relatively small number of patients and as a result data that are not statistically significant. That is why many old drugs with a well-known mechanism of action are re-proposed for the treatment of COVID-19. As no vaccine against COVID_19 has been approved yet, vaccine types have not been included in this paper. In addition, this review did not consider the cases of special patients such as the pediatric population and pregnant women, as they are excluded from clinical trials for ethical reasons.

The strength of this review is providing of the recent data with regard to the management of the COVID-19, within the environment in which information is rapidly changing and being made available; and it will be beneficial to health professionals.

## Conclusion

The ongoing SARS-CoV-2 associated COVID-19 pandemic is continuously emerging worldwide and signifying the greatest spotlight on public health, education, travels, and economic conditions in the current world. The swiftness and dimensions of emerging therapeutic interventions hurled to explore potential treatments for COVID-19 highlight both the necessity and competence to produce superior evidence even at the time of a pandemic. Still, there is no single specific therapy that may give effective responses toward COVID-19. We believe, this paper will be able to provide sufficient information regarding the current treatment strategies and future directions for the pandemic SARS-CoV-2 infection.

Drug treatment is individualized according to the patient’s symptoms. The patient receives adequate care to relieve and treat symptoms. Patients suffering from serious illnesses and complications (such as pneumonia, severe respiratory problems, diabetes, cancer, cardiovascular disease) receive optimal care to support vital functions. Throughout this period, since the beginning of the pandemic, drugs already used in other diseases have been used, the safety of which has already been tested on humans. However, there is a drug that has already been tested for the treatment of people infected with Ebola and MERS, Remdesivir and seems to be an option for a treatment that is available globally.

In spite of the pandemic condition, we cannot forsake the prerequisite for well-designed clinical trials. Therefore, the current situation highlights the urgency for adhering to clinical pharmacology and model-informed drug development to optimize COVID-19 therapies, designing adaptive solidarity trials to decrease therapeutic dilemmas in clinical trial settings, as well as implementing the right patient, right drug, right dosage, and right timing approach to maximize trial success. Finally, adaptive designs for COVID-19 will lead to the development of more vigorous infectious disease research infrastructure and funding to help mitigate future pandemics.

## Author Contributions

CS, MMo, MTI: conceptualization. MMa, AOD, and MTI: validation investigation. DC, AOD, JS-R, and MMa: resources. MMo, AOD, MMa, AM, DC, and JS-R: data curation. MMa, DC, and JS-R: review and editing. All authors: writing. All authors contributed to the article and approved the submitted version.

## Conflict of Interest

The authors declare that the research was conducted in the absence of any commercial or financial relationships that could be construed as a potential conflict of interest.
